# Dicalcin suppresses invasion and metastasis of mammalian ovarian cancer cells by regulating the ganglioside-Erk1/2 axis

**DOI:** 10.1038/s42003-023-05324-w

**Published:** 2023-10-06

**Authors:** Naofumi Miwa, Mayu Hanaue, Kayo Aoba, Ryohei Saito, Ken Takamatsu

**Affiliations:** 1 Department of Physiology, School of Medicine, Saitama Medical University, 38 Moro-hongo, Moroyama, Iruma-gun, Saitama, 350-0495 Japan; 2 Department of Physiology, School of Medicine, Toho University, 5-21-16 Omori-nishi, Ota-ku, Tokyo, 143-8540 Japan

**Keywords:** Metastasis, Peptides

## Abstract

Metastasis, a multistep process including cancer cell migration and invasion, is the major cause of mortality in patients with cancer. Here, we investigated the effect of dicalcin, a Ca^2+^-binding protein, on the invasion and metastasis of ovarian cancer (OC) cells. Extracellularly administered dicalcin bound to the membrane of OV2944 cells, mouse OC cells, and suppressed their migration in vitro; however, cell viability or proliferation were unaffected. Repeated intraperitoneal injection of a partial peptide of dicalcin (P6) prolonged the survival, and reduced the number of microcolonies in the livers of cancer-bearing mice. P6 bound to the ganglioside GM1b in a solid-phase assay; treatment with P6 inhibited the constitutive activation of Erk1/2 in OC cells, whereas excess administration of GM1b augmented Erk activity and cancer cell migration in vitro. Thus, dicalcin, a novel suppressor of invasion and metastasis of OC cells, acts via the GM1b-Erk1/2 axis to regulate their migration.

## Abstract (web summary)

The Ca^2+^-binding protein dicalcin suppresses cell migration and reduces metastasis in cancer-bearing mice.

## Introduction

Dicalcin, a non-enzyme Ca^2+^-binding protein, binds to a zona pellucida (ZP) glycoprotein, a constituent of the extracellular egg-coating envelope, in the presence of Ca^2+^ and regulates the orientation pattern of the filaments as well as the viscoelasticity of the entire envelope, thereby suppressing sperm-egg interaction^1–3^. Dicalcin (also known as S100A11^4^) was originally identified in the cilia of olfactory epithelium, and also found in other tissues, including lung and oviduct^4,5^. In addition to intracellular localization, dicalcin is expressed in extracellular structures such as the egg-coating envelope, suggesting that upon its release from cells, it is retained within the extracellular space by binding to extracellular targets such as the ZP glycoprotein in the egg-coating envelope. Based on this, we hypothesized that dicalcin influences glycoprotein- or oligosaccharide-involved cellular events besides fertilization. Since the processes of invasion and metastasis of cancer cells are known to involve glycoproteins and oligosaccharides^6–9^, we here investigated the potential effect of dicalcin on invasion and metastasis in vitro using human and mouse ovarian cancer (OC) cell lines and in vivo using xenograft mouse model of OCs. In this study, we characterized the suppressive action of dicalcin, and uncovered the signaling mechanisms of dicalcin to regulate the cell migration of OC cells.

## Results

### Dicalcin, a novel suppressor of in vitro invasion of mammalian ovarian tumor cells

**Fig. 1:**
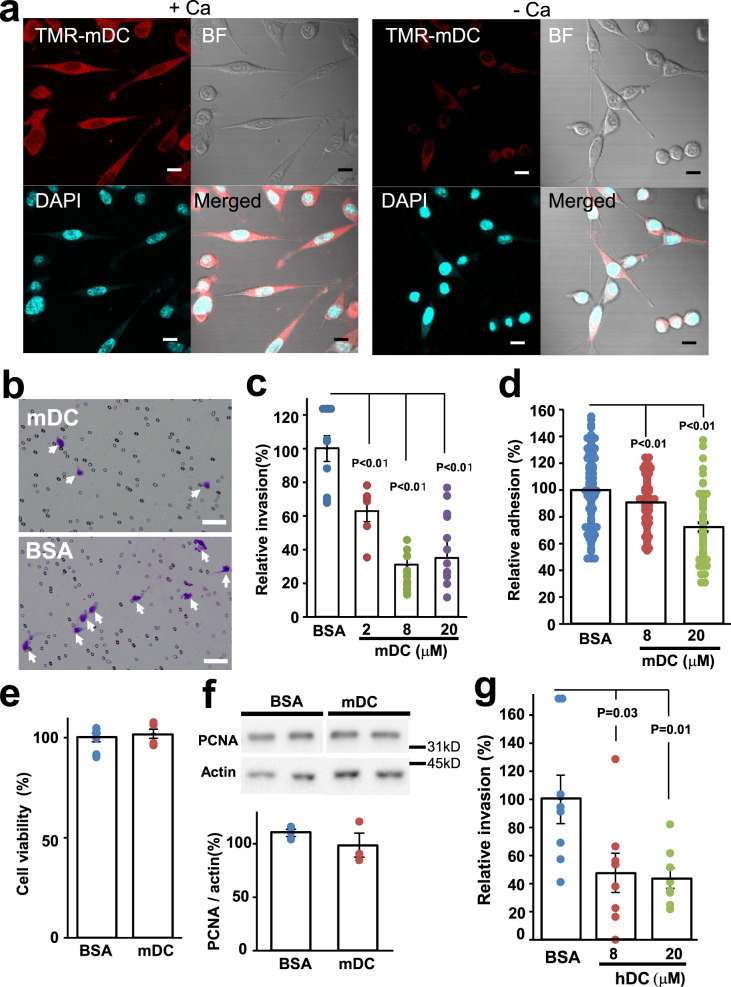
Inhibition of in vitro invasion of mouse and human ovarian tumor cells by dicalcin. **a** Binding of exogenously administered dicalcin to OV2944 cells. TMR-labeled dicalcin bound to OV2944 cells in the presence of 1 mM Ca^2+^ (+Ca), but not in the presence of 3 mM EGTA (−Ca). Scale: 10 μm. **b** In vitro invasion assay. Upper: Representative images of OV2944 cells that migrated through Matrigel-coated membrane, followed by pretreatment with 20 μM mouse dicalcin (mDC). Lower: In vitro invasion assay of OV2944 cells pretreated with bovine serum albumin (BSA). Scale: 50 μm. **c** In vitro invasion assay. Exogenously administered dicalcin suppressed in vitro invasion of OV2944 cells in a dose-dependent manner. The invasion activity for BSA treatment was set to 100% and the data were normalized. The bar graph shows mean data (*n* = 6–13, mean ± s.e.m). Circles show individual data. *p* values represent unpaired Student’s *t-*test. **d** In vitro adhesion assay of OV2944 cells. Pretreated OV2944 cells with dicalcin were loaded on the plate, and cells on the plate were fixed and stained. After washing with phosphate buffer saline (PBS) to remove unattached cells, the number of cells were counted and normalized. Exogenously administered dicalcin suppressed in vitro adhesion of OV2944 cells to the matrix in a dose-dependent manner. The bar graph shows mean data (*n* = 64–105, mean ± s.e.m). Circles show individual data. *p* values represent unpaired Student’s *t-*test. **e** Effect of dicalcin on the cell viability. OV2944 cells following treatment with 10 μM dicalcin or BSA were used for MTT assay. The fluorescent data (OD_560_) was normalized and evaluated. The bar graph shows mean data (BSA, *n* = 10; mDC, *n* = 6; mean ± s.e.m). Circles show individual data. **f** Effect of dicalcin on the cell proliferation. OV2944 cells following treatment with 10 μM dicalcin or BSA were analyzed by western blot using an anti-PCNA antibody. The bar graph shows mean data of the intensities of the blots (*n* = 3, mean ± s.e.m). Circles show individual data. **g** In vitro invasion assay of OVCAR-3 cells. Exogenously administered human dicalcin (hDC) suppressed in vitro invasion of OVCAR-3 cells in a dose-dependent manner. The invasion activity for BSA treatment was set to 100% and the data were normalized. The bar graph shows mean data (*n* = 8, mean ± s.e.m). Circles show individual data. *p* values represent the results of the unpaired Student’s *t-*test.

We first examined the binding of exogenous mouse dicalcin (mDC) to OV2944 cells (mouse OC cells). Bacterially expressed mDC was labeled with tetra-methylcarboxyrhodamine (TMR), a fluorescent dye, and then incubated with OV2944 cells. As shown in Fig. 1a, TMR-labeled mDC (TMR-mDC) bound to OV2944 cells in the presence of Ca^2+^ (+Ca) and this binding was abrogated by the addition of EGTA to the medium (−Ca), indicating that the binding of mDC to OV2944 cells is Ca^2+^-dependent due to its Ca^2+^-dependent conformational change^10^. Furthermore, an anti-dicalcin antibody showed no immunoreactivity with OV2944 cells, indicating the lack of endogenous dicalcin within OV2944 cells; hence, the action of endogenous dicalcin was not detected in the following experiments (Supplementary Fig. 1). We next investigated whether exogenously administered mDC affects the invasivity of OV2944 cells. Results of in vitro invasion assay using Matrigel showed that pretreatment of OV2944 cells with mDC significantly reduced its invasion in a dose-dependent manner (Fig. 1b, c). Moreover, pretreatment of OV2944 cells with mDC significantly decreased the ratio of OV2944 that adhered onto the Matrigel matrix (Fig. 1d). Dicalcin treatment neither affected cell viability nor proliferation as revealed by the 3-(4,5-dimethylthiazol-2-yl)-2,5-diphenyltetrazolium bromide (MTT) assay and western blot analysis using an anti-proliferating cell nuclear antigen (PCNA) antibody, respectively (Fig. 1e, f). We further investigated the effect of dicalcin on OVCAR-3 cells (human OC cells). Similar to OV2944 cells, TMR-labeled human dicalcin (hDC) bound to OVCAR-3 cells in the presence of Ca^2+^ (Supplementary Fig. 2a), and incubation of OVCAR cells with hDC reduced cell invasion in a dose-dependent manner (Fig. 1g). Treatment with hDC did not affect the cell viability of OVCAR-3 cells (Supplementary Fig. 2b). These results demonstrated that dicalcin is a novel suppressor of in vitro invasion of human and mouse OC cells.

### Identification of amino acid regions responsible for suppressing metastasis of OV2944 cells

**Fig. 2:**
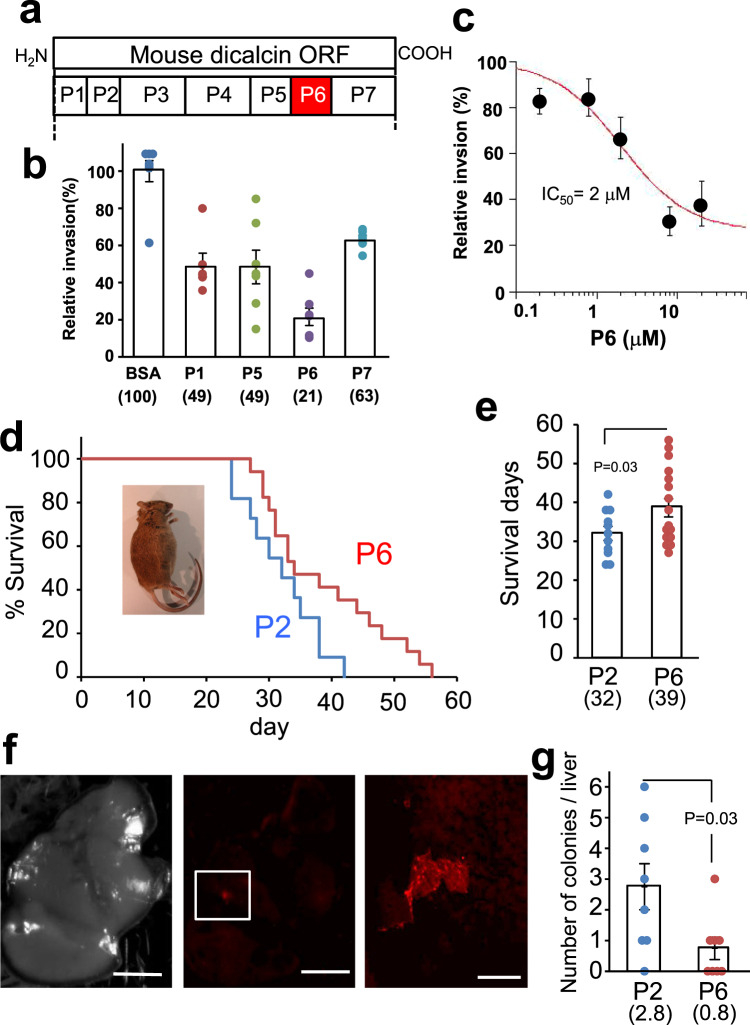
Identification of the amino acid region that is responsible for the suppressive action of dicalcin on OV2944 invasivity. **a** Mapping of partial amino acid region and synthetic peptides. The primary sequence of mouse dicalcin was divided into seven regions. Binding activities of seven regions of dicalcin to OV2944 cells were examined. The amino acid region that corresponds to the peptide with the most intense binding is highlighted. **b** In vitro invasion assay using synthetic peptides. Synthetic peptides bearing higher binding activities (P1, P5, P6, P7) were examined for in vitro invasion assay using OV2944 cells. Exogenously administered P6 showed a robust suppression of in vitro invasion. Numbers in the parentheses represent averaged values in each condition. The bar graph shows mean data (*n* = 6–8, mean ± s.e.m). Circles show individual data. **c** Dose-dependency of suppressive effect of P6 on in vitro invasion. Exogenously administered P6 exhibits a dose-dependent suppression of in vitro invasion of OV2944 cells (*n* = 10–20). **d** Survival rate of tumor-transfected mice. B6C3F1 mice were intraperitoneally injected with OV2944 cells (1 × 10^5^ cells) that were pretreated either with 10 μM P6 or P2. Following cell injection, mice were intraperitoneally injected with the appropriate peptide until the end of the study (injection dose and schedule: 3 nmol/150 μl/2 days, Supplementary Fig. 15). The survival of transfected mice was evaluated by Kaplan–Meier analysis. Inset photo: sacrificed mice developed a large quantity of ascites. **e** Averaged survival days of cancer-bearing mice. Numbers in the parentheses represent averaged values in each condition. The bar graph shows mean data (P2, *n* = 11; P6, mean ± s.e.m., *n* = 17, unpaired Student’s *t-*test). Circles show individual data. **f** Reduced micrometastasis in P6-treated mice. Mice were *i.p*. injected with OV2944 cells expressing tdTomato and repeatedly injected with the appropriate peptide. After 20 days, cell-injected mice were sacrificed; the location of metastasis in the liver was observed with in vivo imaging microscope (Left, bright field image of the liver, Scale, 5 mm; Middle, fluorescent image of the liver, Scale, 5 mm; Right, enlarged image of the colony indicated in the square of the middle, Scale, 1 mm). **g** The average number of colonies was decreased in P6-treated mice, compared with that for P2-treated mice. Numbers in the parentheses represent averaged values in each condition. The bar graph shows mean data (mean ± s.e.m., *n* = 8, unpaired Student’s *t-*test). Circles show individual data.

We observed that the suppressive action of mDC requires its binding to OV2944 cells. To map the binding region of mDC, we synthesized rhodamine-labeled peptides encompassing the entire sequence of mDC (dcp1-dcp7 in Fig. 2a and Supplementary Fig. 3) and examined their binding to OV2944 cells. Among the peptides examined, four peptides (P1, P5, P6, and P7) showed substantial binding to OV2944 cells (Supplementary Figs. 4–10). Since P2 showed no apparent binding to the cells, we considered that exogenous treatment of P2 was an appropriate control treatment in the subsequent peptide-administered experiments. To identify the sequences accounting for the mDC anti-migratory effects, we examined the effects of these four peptides on in vitro invasion of OV2944 cells. Our results showed that treatment with P6 led to maximum suppression of the cell invasion (21% of control, *p* < 0.01, unpaired Student’s *t-*test; Fig. 2b), and the suppression occurred in a dose-dependent manner with an IC_50_ of 2 μM and a Hill coefficient of 0.9 (Fig. 2c). The 3-D modeling of dicalcin structure^2,10^ revealed that the region spanned by P6 is dominated by an α-helix and a loop and present on its molecular surface (Supplementary Fig. 11). The hDC-derived peptide (hDC-P6) that corresponds to mouse P6 bound to OVCAR-3 cells, and suppressed in vitro invasion of OVCAR-3 cells in a dose-dependent manner (Supplementary Fig. 12). Accordingly, we inferred that this region is essential for the dicalcin action. We examined whether P6 exerted suppressive effects on other tumor cells, and found that hDC-P6 bound to PC-3 cells, human prostate cancer cells (Supplementary Fig. 13a) and treatment of PC-3 cells with P6 significantly suppressed in vitro invasion (Supplementary Fig. 13b). In addition, we examined the effect of dicalcin on the migration of normal ovarian epithelial cells using T-Ag-MOSE cells (SV40-infected immortal mouse normal ovarian epithelial cells). P6 showed no apparent binding to the cells; therefore, it showed no effect on the migratory activity (Supplementary Fig. 14). These results suggested that dicalcin exerts a suppressive action on the cancer cells.

We next examined the effect of dicalcin on survival of cancer-bearing mice. B6C3F1 mice were intraperitoneally injected with OV2944 cells (1 × 10^5^ cells) to create a xenograft model that mimics metastasis of OC within the intraperitoneal cavity. The injected OV2944 cells were pretreated either with P6 or P2 as a control (10 μM each). Following cell injection, mice were intraperitoneally injected with the appropriated peptide (3 nmol/150 μl) repeatedly once every 2 days until the animals died, and the survival rate of the treated mice was determined. Notably, the dead mice had severe ascites (inset photo in Fig. 2d). Kaplan–Meier analysis revealed that repeated injection of P6 significantly prolonged the survival of the tumor-injected mice (Fig. 2d, *p* < 0.03, *n* = 30). The averaged survival days for the OV2944 injected mice increased to ~39 days in P6-treated mice, compared with ~32 days in P2-treated mice (Fig. 2e). The increase in survival days (*i.e*. 20%) was comparable with previous data obtained for paclitaxel (TXL, a common therapeutic agent for several types of cancers)^11^; however, the dose of P6 used in our study was significantly lower than that of TXL (~1/60 of the TXL amount), demonstrating the biological and clinical impact of P6 as a leading compound for anti-metastasis. To further characterize the effect of P6 on metastasis, we intraperitoneally injected OV2944 cells expressing the fluorescent protein tdTomato into B6C3F1 mice and examined the effect of peptide treatment on micrometastasis in the liver. Briefly, OV2944 cells expressing tdTomato were collected by flow cytometry (Supplementary Fig. 15), and pretreated either with P6 or P2 (10 μM each) prior to the injection. Following cell injection, mice were intraperitoneally injected with the peptide repeatedly once every 2 days. Twenty days after cell injection, mice were sacrificed, and their livers were analyzed. Micrometastatic colonies were observed in the livers of mice from both groups (Fig. 2f). However, the number of colonies was significantly lower in mice treated with P6 (0.8 for P6-treated mice, 2.8 for P2-treated mice, Fig. 2g). Thus, these results showed that the amino acid region corresponding to the P6 peptide region is responsible for suppressing OV2944 cell metastasis.

### Dicalcin-derived peptide suppresses the OV2944 migration by downregulating Erk1/2 activity

**Fig. 3:**
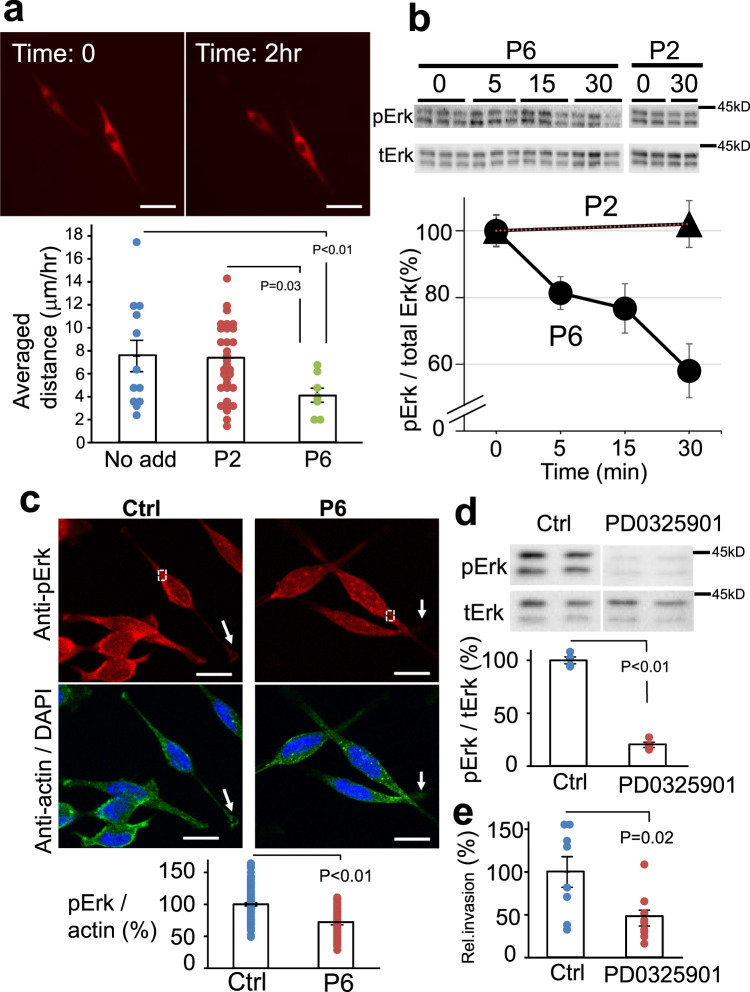
Dicalcin-derived peptide inhibits the OV2944 migration by downregulating Erk1/2 pathway. **a** Top: Representative time-lapse imaging of OV2944 cells expressing tdTomato at time 0 and 2 h later. Bottom: The migration distance of OV2944 cells. The migration activity of OV2944 cells was decreased in the presence of P6, compared with that for nothing complemented (no add), and for P2 (Ctrl). The bar graph shows mean data (mean ± s.e.m., *n* = 11–30). Circles show individual data. *p* values represent unpaired Student’s *t-*test. Scale: 50 μm. **b** Addition of P6 into the medium suppressed the Erk1/2 activity of OV2944 cells. Top: Following peptide treatment, cell extracts were prepared at the indicated times and a portion of them was subjected to western blot to quantitate the phosphorylation of Erk. Bottom: The relative Erk activity (pErk/total Erk) was plotted as a function of time (*n* = 8, mean ± s.e.m.). **c** Representative confocal images of pErk- and actin-staining. Following 30-min treatment either with P6 (P6) or P2 (Ctrl), OV2944 cells were fixed and double-stained with anti-pErk (red) and anti-actin (green) antibodies. The immunoreactive intensities of pErk in the cytosol were quantified (designated as square in **a**, 10 µm^2^ per square) and calibrated by the immuno-intensity for actin at the identical square. The ratio of pErk+/actin+ for control was set to 100% and the data were normalized. The graph shows mean data (a.u., optical arbitrary units; Ctrl, *n* = 94; P6, *n* = 89; mean ± s.e.m). Circles show individual data. Addition of P6 suppressed pErk immunoreactivity (~65% of control). We also note that the pErk immunoreactivity in the lamellipodia disappeared in the P6-treated cells (arrows). Scale: 10 μm. **d** Effect of MEK inhibitor treatment on pErk signal. OV2944 cells were treated with MEK inhibitor (PD0325901, 1 µM). After 30-min treatment, cell extracts were prepared and a portion of them was subjected to western blot. The ratio of pErk/tErk for control was set to 100% and the data were normalized. The graph shows mean data (*n* = 3, mean ± s.e.m.). Circles show individual data. **e** In vitro invasion assay for MEK inhibitor. OV2944 cells were treated with 1 µM PD0325901 for 15 min, and the invasivity of the cells was evaluated in in vitro invasion assay. The graph shows mean data (*n* = 8, mean ± s.e.m,). Circles show individual data.

How does dicalcin suppress metastasis of OV2944? Metastasis involves several steps, including invasion, angiogenesis, adhesion, and proliferation. As shown in Fig. 1, treatment with dicalcin had no effect on cell viability or proliferation of OV2944 cells (Fig. 1e, f). Next, we examined the effect of dicalcin on the migration of OV2944 cells by time-lapse imaging of tdTomato-expressing OV2944 cells to measure the single-cell migration. This imaging revealed that their migratory activity significantly decreased in the P6-containing medium, compared with that in the presence of P2 (4.1 μm/h for P6, 6.9 μm/h for P2, and 7.5 μm/h for no addition, Fig. 3a), indicating that P6 suppressed the migration of OV2944 cells. Extracellular signal-regulated kinase 1/2 (Erk1/2) signaling pathway is one of the major signaling pathways activated during cell migration^12–15^. Our western blot analysis showed that OV2944 cells exhibit constitutive activation of Erk1/2 (i.e., the higher level of phosphorylated Erk; pErk). P6 (10 μM) reduced the level of pErk to ~55% of the basal level following 30-min treatment (Fig. 3b). Immunohistochemical study confirmed Erk activation in the cell body of the control OV2944 cells as well as at the terminal of cell protrusions; they are probably equivalent to invadopodia or lamellipodia (arrows in Ctrl in Fig. 3c). However, treatment with P6 significantly reduced pErk levels (~65% of control, Fig. 3c), and pErk signals at the invadopodia diminished in the P6-treated OV2944 cells (arrows in P6 in Fig. 3c). To confirm the involvement of Erk1/2 in OV2944 migration, we examined the effect of Erk1/2-pathway inhibition with PD0325901, a mitogen-activated protein kinase kinase (MEK) inhibitor^16^. Treatment with PD0325901 (1 μM) abolished intracellular Erk1/2 activity (Fig. 3d) and suppressed migration (Fig. 3e). These results indicated that P6 suppresses the OV2944 migration by downregulating Erk1/2 activity. We also examined the effect of P6 administration on Erk1/2 activity of OVCAR-3 cells (human OC cells), and confirmed that P6 reduced the level of Erk1/2 activity to ~60% of that in the control (Supplementary Fig. 16a). Furthermore, we examined effect of P6 administration on the activities of other signaling molecules such as p38 mitogen-activated protein kinase (MAPK) and AKT kinase. Our western blot analysis demonstrated that p38 MAPK activity (i.e., the level of phospho-p38 MAPK) was negligible and that significant contribution of p38 MAPK in OVCAR-3 cells was unlikely (Supplementary Fig. 16b). Furthermore, P6 administration did not affect the level of AKT kinase (Supplementary Fig. 16c). These results suggested that DC affects Erk1/2-activity uniquely among signaling pathways that we examined in the OVCAR-3 cells.

### Dicalcin-derived peptide binds to a ganglioside of the OV2944 cell membrane to regulate Erk signaling

**Fig. 4:**
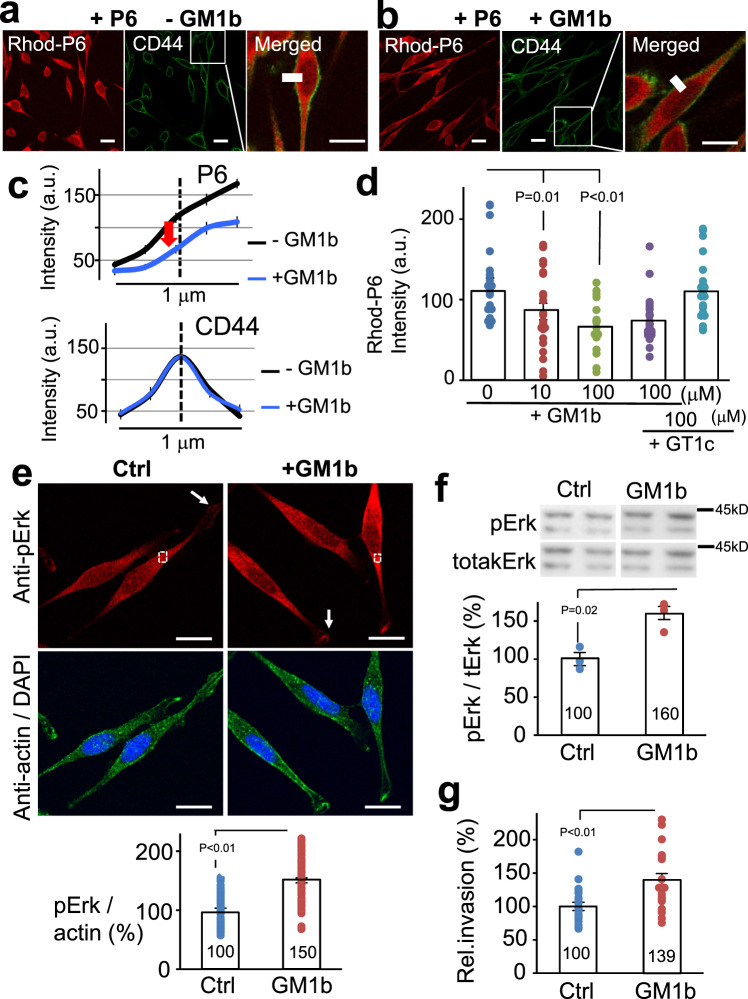
Dicalcin-derived peptide interacts with GM1b on the membrane of OV2944 cells. **a** Representative confocal images of OV2944 cells treated with P6. OV2944 cells were treated with rhodamine-labeled P6 (Rhod-P6) and anti-CD44 antibody as a marker of the membrane (CD44). Scale: 10 μm. **b** Representative confocal images of OV2944 cells treated both with P6 and GM1b. OV2944 cells were treated with Rhod-P6 and anti-CD44 antibody, together with GM1b. Scale: 10 μm. **c** GM1b reduced the fluorescent signal of rhodamine-P6 in OV2944 cells. Fluorescent signals of P6 and CD44 were quantified across the membrane (white line in **a** and **b**; *n* = 20–30, mean ± s.e.m., a.u. optical arbitrary units). Note that the peak signal of CD44 indicates the site of the membrane of OV2944 cells. The signal intensity for P6 at the cell membrane was decreased in the presence of GM1b (P6, +GM1b). **d** Rhodamine-P6 signals were compared among the indicated conditions of treatments. OV2944 cells were treated with P6 peptide (5 μM) in the presence or absence of GM1b or GT1c at indicated concentrations. GM1b inhibited the binding of P6 to OV2944 cells in a dose-dependent manner, whereas GT1c did not lead to significant changes. The graph shows mean data (*n* = 18–26, mean ± s.e.m.). *p* values represent two-sided unpaired Student’s *t-*test. Circles show individual data. **e** Representative images of pErk- and actin-staining after 30-min incubation either of GM1b (GM1b) or glucose (Ctrl). OV2944 cells were fixed and double treated with anti-pErk (red) and anti-actin (green) antibodies. Immunoreaction intensities in the cytosol of the cells were quantified (designated as square in **a**, 10 µm^2^ per square), calibrated by the actin-intensity at the identical square, and resultant values were analyzed (a.u. optical arbitrary units). The addition of GM1b increased pErk immunoreactivity (150% of control, Ctrl, *n* = 63; +GM1b, *n* = 72), indicating GM1b-dependent augmentation of Erk activity within cells. Numbers in the bars represent averaged values in each condition. *p* values represent two-sided unpaired Student’s *t-*test. Scale: 10 μm. **f** Addition of GM1b into the medium augmented the Erk1/2 activity of OV2944 cells. Top: Cell extracts were prepared 30-min after the addition of GM1b into the culture medium and a portion of them was subjected to western blot analysis. Bottom: The ratio of pErk/tErk for control was set to 100% and the data were normalized. The graph shows mean data (*n* = 4, mean ± s.e.m.). Circles show individual data. Numbers in the bars represent averaged values in each condition. *p* values represent two-sided unpaired Student’s *t-*test. **g** Increased migratory activity by addition of GM1b. OV2944 cells were treated with GM1b (50 μM) for 15 min, and the migratory activity of the cells was evaluated by in vitro invasion assay. The graph shows mean data (*n* = 21, mean ± s.e.m.). Circles show individual data. *p* values represent two-sided unpaired Student’s *t-*test. Numbers in the bars represent averaged values in each condition.

Since extracellular administration of dicalcin suppressed the invasivity of OV2944 cells, we hypothesized that the target molecules for dicalcin are probably localized on the cell membrane. We sought the target molecules for dicalcin among the membrane proteins and lipids present in OV2944 cells, but failed to identify the candidates initially. Instead, using a solid-phase assay, we found that P6 peptide strongly bound to oligosaccharides present in two gangliosides, namely GM1b and GT1c (Supplementary Fig. 17a). Gangliosides, the sialic acid-containing glycosphingolipids, localizes in the outer leaflet of the plasma membrane of vertebrate cells. They comprise the core lipids of the micro domain referred to as lipid rafts that are enriched in signaling molecules, including a variety of receptors, ion channels, and kinases^17^ (Supplementary Fig. 17b). By forming a complex with these molecules, gangliosides transduce extracellular cues into intracellular signals and play various functional roles in cell proliferation and cell-cell interactions. For example, the ganglioside GM1 binds to TrkA and enhances the TrkA signal in response to NGF stimulation, resulting in better cellular differentiation^18,19^. We examined whether dicalcin binding to OV2944 involves GM1b or GT1c present on the cell surface. Exogenously administered P6 bound to the membrane of the cells (Fig. 4a, b and Supplementary Fig. 18). Exogenously administered GM1b significantly prevented the binding of rhodamine-labeled P6 (Rhod-P6) to the membrane of the cells (arrow in Fig. 4c). Moreover, the degree of prevention depended upon the amount of GM1b added (Fig. 4d). In contrast, the administration of GT1c showed no effect on P6 binding to the membrane of OV2944 cells (Fig. 4d), suggesting that GT1c is not involved in P6 binding to the cell surface in the case of OV2944 cells. Furthermore, we treated cells with neuraminidase to eliminate the terminal sialic acid residue of GM1b, and examined the binding of Rhod-p6; treatment with neuraminidase reduced the binding of Rhod-P6 (Supplementary Fig. 19). Based on these data, we hypothesized that if endogenous GM1b is the target of P6, the addition of excess GM1b should reverse the action of dicalcin and enhance Erk activation and migratory activity. Indeed, GM1b (50 μM) treatment increased the level of immunohistochemical staining for pErk (~150% of control, Fig. 4e) as well as the level of pErk, as evidenced by western blot analysis (~160% of control, Fig. 4f). In addition, treatment with GM1b augmented the migratory activity of OV2944 cells (~140% of control, Fig. 4g). Furthermore, we treated the cells either with GM1b alone or GM1b together with PD0325901, and found that combined administration of GM1b and PD0325901 canceled the GM1b-induced upregulation of Erk1/2, indicating that GM1b-induced signaling converges onto the Erk1/2 activation pathway (Supplementary Fig. 20). Collectively, these results suggested that GM1b is the endogenous target of dicalcin, at least in OV2944 cells, and that P6 binding to GM1b downregulates Erk signaling.

## Discussion

Our study outcomes showed that the extracellularly administered dicalcin (P6 to be more specific) binds to the GM1b ganglioside present in the membranes of OV2944 cells, leading to the downregulation of constitutively activated Erk1/2 and subsequent suppression of their migration, which ultimately inhibits local invasion and metastasis of OV2944 cells (for a schematic model, see Supplementary Fig. 21). Therefore, the initial step in the dicalcin action is its binding to GM1b in cancer cell membrane. Certain membrane gangliosides bind to a variety of growth receptors, including the epidermal growth factor receptor, insulin-like growth factor receptor, and the NGF receptor, modulating downstream signaling and biological functions^20^. For example, exogenous GM3 (another ganglioside) activates MAPK in neuroblastoma cells and affects neuritogenesis^21^, consistent with the exogeneous GM1b-dependent augmentation of Erk1/2 activity in OV2944 cells observed in our study (Fig. 4e, f). GM1b is expressed in membranes of several types of cancer cells, including HH870 (prostate cancer)^22^, Y79 (retinoblastoma)^23^, and YAC-1 cells (lymphoma)^24^. In addition, downregulated GM1b expression inhibits invasivity in vitro and tumor growth in vivo^25^. Therefore, we propose that the dicalcin-GM1b-Erk1/2 axis is involved in the dicalcin-dependent suppression of OC cell migration. Furthermore, we found that dicalcin suppressed the migration of OC cells (OV2944 and OVCAR-3) as well as prostate cancer cells (PC-3) in vitro (Fig. 1 and Supplementary Fig. 13). Given that cancer-related pathways are shared among different cancers, we consider that the dicalcin-GM1b-Erk1/2 axis may also be shared among some cancer types that express GM1b and Erk1/2. Among the gangliosides examined, P6 exhibited preferential binding to GM1b and GT1c. However, P6 binding to other gangliosides, GM1a and GD1a with sugar moieties (i.e., -Glu-Gal-GalNAc) identical to those in GM1b, was comparable to the levels observed in the control, suggesting that P6 interacts with the supramolecular structure of sialylated GM1b and GT1c, and not with the sole structure of the core sugar. The detailed mechanisms of how P6 binding to the GM1b is transduced to GM1b-coupled membrane proteins and how dicalcin and GM1b interact (for example, the interaction surface and stoichiometry etc.) remain to be characterized.

Our results clearly demonstrate that treatment with P6 decreases Erk1/2 activity throughout the cell body, particularly in the terminal of the lamellipodia (Fig. 3), whereas the addition of GM1b reverses this effect (Figs. 3 and 4). Erk1/2 is a prototypic MAPK that is activated in response to growth factors and the extracellular matrix via binding to cognate receptors and integrins, respectively. Activated Erk phosphorylates a variety of proteins, including transcription factors, and downstream kinases such as p90 ribosomal S6 kinase (RSK), thereby regulating cell proliferation, survival and motility^26^. Pretreatment with P6 suppressed in vitro migration of OV2944 cells, though it had no effect on cell proliferation or survival (Fig. 1). In rat glioma cells, cellular migration and proliferation are mutually exclusive cellular events^27^, which accounts for a decreased proliferation rate in invasive glioma cells^28^. We speculate that the dicalcin-GM1b-Erk1/2 axis may function only when cancer cells turn metastatic. The intracellular signaling to facilitate metastasis is triggered by extracellular clues that converge into various downstream effector pathways, including actin polymerization and actomyosin contractility. It remains to be determined whether and how dicalcin regulates the intracellular migratory machinery. It also remains to be addressed whether ERK1/2 is the only mechanism whereby dicalcin is mediating its anti-tumor effects.

We believe that dicalcin offers at least two advantages as a therapeutic candidate for OC. First, it effectively suppresses the migration of OC cells. The current strategies for developing therapeutic agents for OC have focused mostly on inhibiting the clonal proliferation of cancer cells^29^. Although we agree that suppressing cellular proliferation is important, we also believe that the potential of inhibiting local invasion and metastasis of cancer cells as a long-term therapeutic strategy should not be underestimated. In this regard, dicalcin was shown to suppress the migration of different cancer cell types in vitro (OC cells and prostate cancer cells, Fig. 1 and Supplementary Fig. 13) and repeated intraperitoneal administration of dicalcin prolonged the survival of cancer-bearing animals (Fig. 2), which provides a strong evidence for the therapeutic potential of dicalcin for the OC. The second advantage is the water solubility of this peptide. Intraperitoneal chemotherapy with TXL is currently a standard therapy for the treatment of OC^30^. However, TXL has poor water solubility, and hence preparation of an oil-in-water emulsion is necessary prior to intravenous administration, which limits the concentration of the TXL to ~10 μg/ml or less. Although some supplements have been introduced to improve efficacy such as cholesterol^31^ or dextrose^32^, the intravenous administration of TXL is mandatory for favorable clinical outcomes. P6 used in our study is expected to be acidic (pI = 3.0) with higher water solubility, giving it a clear advantage over TXL. Furthermore, the dose of P6 used in our in vivo metastasis analysis was significantly lower than that of TXL used in a previous report (P6, 0.1 mg/kg/day; TXL, 5.7 mg/kg/day^8^), suggesting that P6 may be an effective therapeutic agent for OC.

In conclusion, our results demonstrate the novel suppressive effects of dicalcin on migration and metastasis of OC cells using in vitro and in vivo experimental models. Dicalcin bound to mouse OC cells via binding to GM1b present in the cell membrane. A partial peptide P6 derived from dicalcin downregulated Erk1/2 activity, thereby suppressing cancer cell migration. Repeated intraperitoneally injection of P6 also inhibited metastasis of OC cells in vivo and prolonged the averaged survival of cancer-bearing mice. We believe that our study provides novel insights into the molecular machinery underlying the metastasis in OC cells and may lead to the development of potent, bioactive compounds capable of inhibiting cancer metastasis.

## Materials and methods

### Animals

All animal experiments were approved and conducted in accordance with the animal care committee’s at Saitama Medical University and Toho University. We have complied with all relevant ethical regulations for animal testing. For in vivo invasion and micrometastasis assays, female mice (B6C3F1, 20–58 weeks old) were used.

### Expression of mouse and human dicalcin in *E. coli*

Recombinant proteins of mouse and human dicalcin were prepared according to procedures shown previously^9^. The full-length coding regions of mouse and human dicalcin were PCR-amplified, ligated with pET-3a (Novagen, EMD Biosciences, San Diego, CA) and introduced into *E. coli* BL21 pLysS (Novagen). Recombinant proteins were purified according to procedures as described previously^4^. The sequences of mouse and human dicalcin are as follows: mouse dicalcin, NP_058020; human dicalcin, NP_005611.

### Fluorescently labeling of dicalcin

Purified recombinant dicalcin was labeled with a fluorescent dye, tetramethylrhodamine (TMR), according to the manufacturer’s manual (protein labeling kit, Olympus, Japan) and dialyzed against Tris-buffered saline (TBS). Labeling of dicalcin was confirmed using a fluorescent image analyzer (Typhoon 9400; GE Health Care, Piscataway, NJ).

### Cell culture

The mouse and human ovarian cancer cell lines (mouse, OV2944; human, OVCAR-3) as well as human prostate cancer cell line (PC-3) were obtained from Riken Bioresource (Tsukuba, Japan); grown in DMEM (Thermo Fisher Scientific) with 10% fetal bovine serum (FBS), pen/strep, 5% CO_2_, 37 °C.

### In vitro binding of dicalcin and its derivative peptides to OV2944 and OVCAR-3 cells

Cultured cells were fixed in 4% paraformaldehyde. After blocking, cells were treated with fluorescently-labeled dicalcin or its derivative peptides. After rinse with PBS, stained cells were observed using either a conventional microscope (Olympus, Tokyo, Japan) or a confocal laser microscope (LSM510; Carl Zeiss, Oberkochen, Germany) with an appropriate set of excitation and emission filters.

### Adhesion of dicalcin onto Matrigel

Adhesion of cells onto the extracellular matrix was examined by using Matrigel (BD Falcon, Franklin, Lakers, NJ) according to the manufacturer’s protocol. Briefly, certain amount of cells were pretreated with mouse dicalcin (8 and 20 μM), or BSA (20 μM) for 20 min at RT. After rinsing with DMEM, cells were placed on the Matrigel-coated culture plate. An hour later, after rinse with PBS, cells were fixed by 3% paraformaldehyde, stained by Crystal violet; the ratio of the number of stained cells/the number of spreader cells was normalized and evaluated as the index of adhesion.

### In vitro invasion assay

In vitro invasion assay  was performed by using BD BioCoat Matrigel invasion chamber (BD Falcon, Franklin, Lakers, NJ) according to the manufacturer’s protocol. Briefly, certain amount of cells were pretreated with several materials as follows: mouse or human dicalcin, its derivative peptides, BSA at indicated concentrations, or mitogen-activated protein kinase kinase (MEK) inhibitor (1 μM, PD0325901 or Mirdametinib, Chemscene, CS-0062), for 20 min at RT. After rinsing with DMEM, cells were placed on the Matrigel-coated inserts, which were immersed into the well of a 24-well plate that contained DMEM with 10% FBS at 37 °C, 5% CO_2_. After 16 h at 37 °C, the membrane on the upper insert was stripped and stained with crystal violet. The ratio of the number of stained cells/the number of spreader cells was evaluated as the index of invasion activity. This index for control condition was set to 100% and the data were normalized.

### MTT assay

To evaluate cell viability after dicalcin treatment, the 3-(4,5-dimethylthiazol-2-yl)-2,5-diphenyltetra-zolium bromide (MTT) assay was done as described previously^33^. Briefly, MTT was converted from the yellow, water-soluble tetrazolium to the blue, water-insoluble formazan by cellular mitochondrial dehydrogenases. Because the rate of this reaction is proportional to the number of living cells, the amount of blue formazan reflects cell viability. In this assay, cultured cells in 96-well plates were treated with 10 μM dicalcin. Two hours later, 100 μM MTT was added. Twenty-four hours after the addition of MTT, 20% SDS in dimethylformamide was added to dissolve formazan, and OD_560_ was measured.

### In vivo invasion assay

OV2944 cells (1 × 10^5^ cells/mouse) were injected intraperitoneally, and thereafter dicalcin-derived or control peptides (3 nmol/6 μg/150 μl/2 days) was intraperitoneally injected per 2 days until the death. The survival of mice was evaluated by Kaplan–Meier analysis.

### Micrometastasis assay

To obtain fluorescently-detectable cells for in vivo micrometastasis assay, OV2944 cells were transfected with tdTomato (2 μg/100 μl, Invitrogen) using FuGENE HD transfection reagent (Promega). Two days later, cells were dissociated and Tomato-expressing cells (i.e., fluorescently-labeled cells) were isolated using a FACSAria cell sorter (BD Bioscience) into DMEM with 10% FBS. After cell sorting by FACS, OV2944 cells expressing tdTomato (1 × 10^5^ cells/ml; tdTomato, Molecular probe) were injected intraperitoneally, accompanied by dicalcin-derived or control peptides (3 nmol/150 μl/2 days). After 20 days, cell-injected mice were sacrificed; the location of micrometastasis in the liver were observed with in vivo imaging microscope (OV110, Olympus).

### In vitro migration assay

OV2944 cells were transfected with tdTomato similarly as described above. Two days later, they were treated with dicalcin-derived (P6) or control peptides (P2) (final conc: 10 μM) in a 12-well culture plate, observed per hour during ~18 h by using time-lapse imaging microscope (Olympus). The distance of the cell location between successively captured images was measured and analyzed.

### Western blot analysis

Cultured cells were treated either with dicalcin-derived peptide (10 μM), MEK inhibitor (1 μM, PD0325901 or Mirdametinib, Chemscene, CS-0062) or GM1b (50 μM, Elicityl, GLY097). Following treatment during indicated times, cell extracts were prepared and a portion of them was electrophoresed and blotted onto a PVDF membrane (Immobilon P; Millipore, Billerica, MA). After blocking, blots were probed with primary antibodies at 4 °C overnight. After washing, blots were treated with HRP-labeled proper secondary antibodies, and immunoreactive proteins were visualized by LAS-1000 (Fujifilm, Tokyo, Japan). Primary antibodies used here were anti-phospho-Erk1/2 (Cell signaling, 9101, 1:100), anti-Erk1/2 (Santa Cruz, SC-94, 1:5000), anti-phospho Akt (Cell signaling, 4058, 1:100); anti-Akt (Cell signaling, 4691, 1:1000); anti-phospho p38 MAPK (Cell signaling, 9211, 1:100); anti-p38 MAPK (Cell signaling, 9212, 1:1000), anti-actin (Merck, MAB1501, 1:5000), and anti-PCNA (Santa Cruz, SC-56, 1:200) antibodies.

### Immunocytochemistry of cultured cells

Cultured cells were fixed in 4% paraformaldehyde for 10 min at RT. Some cells were pretreated with MEK inhibitor (1 μM, PD0325901) for 15 min at RT prior to preparation. After permeabilizing and blocking, cells were incubated with antibodies at 4 °C overnight. After incubation with secondary antibody, detection and capturing of immunopositive cells were done using either a conventional microscope (Olympus, Tokyo, Japan) or a confocal laser microscope (LSM510; Carl Zeiss, Oberkochen, Germany) with an appropriate set of excitation and emission filters. To visualize nuclei morphology, cells were stained with 1 mg/ml DAPI. Primary antibodies used here were anti-phospho-Erk1/2(Cell signaling, 9101, 1:100), anti-CD44 antibody (abcam, ab25340, 1:100), anti-actin(Merck, MAB1501, 1:500).

### Modeling of three-dimensional structure of dicalcin and gp41

The graphics program RASMOL was used for modeling of three-dimensional structure of dicalcin already reported in our previous study^2,10^.

### Glycan array analysis

Glycan array assay was performed by using a glycan array plate (Sumitomo Bakelite Co., Tokyo, Japan). Biotin-labeled P6 (1 μM) was incubated overnight at 4 °C with the array bearing a wide range of glycans including N-glycans, O-glycans, Lewis glycans, lactosamine, gangliosides, and glucosides. After rinse, the array was treated with Cy3-conjugated streptavidin (Sigma, S6402, 1:1000) and P6-binding glycans were detected by using fluorescence scanner (Arrayscan, Thermo Scientific), and quantitated.

### Neuraminidase treatment

OV2944 cells were treated with neuraminidase (1 μM, 37 °C, 1 h, Fujifilm Wako, Japan) to eliminate terminal sialic acid residue of GM1b. Cells were treated with biotin-labeled *Maackia amurensis* II (MLAII, 50 μg/ml, vector labs, FITC-labeled wheat germ agglutinin (WGA, 50 μg/ml, vector labs, 30 min, RT). MLAII recognized sialic acid, whereas WGA did not. After fixation, cells were treated with Texas-red streptavidin (1 μg/ml, 30 min, RT). Following fixation, cell were treated with rhodamine-labeled p6 (Rhod-P6) and anti-CD44 antibody (Abcam, ab25340, 1:100), and reacted with secondary antibody and mounted.

### Reporting summary

Further information on research design is available in the Nature Portfolio Reporting Summary linked to this article.

### Supplementary information

Supplementary Information

Reporting Summary

## Data Availability

All data supporting the findings of this study are available within the paper and/or the Supplementary Information. Uncropped and unedited blot/gel images are available in Supplementary Figs. 22–27.

## References

[1] Miwa N (2010). Dicalcin inhibits fertilization through its binding to a glycoprotein in the egg envelope in Xenopus laevis. J. Biol. Chem..

[2] Miwa N, Ogawa M, Hanaue M, Takamatsu K (2015). Fertilization competence of the egg-coating envelope is regulated by direct interaction of dicalcin and gp41, the Xenopus laevis ZP3. Sci. Rep..

[3] Hanaue M, Miwa N (2017). Structural and rheological properties conferring fertilization competence to Xenopus egg-coating envelope. Sci. Rep..

[4] Hanaue M (2011). Characterization of S100A11, a suppressive factor of fertilization, in the mouse female reproductive tract. Mol. Reprod. Dev..

[5] Miwa N, Kobayashi M, Takamatsu K, Kawamura S (1998). Purification and molecular cloning of a novel calcium-binding protein, p26olf, in the frog olfactory epithelium. Biochem. Biophys. Res. Commun..

[6] Variki A (1993). Biological roles of oligosaccharides: all of the theories are correct. Glycobiology.

[7] Hakomori S (2002). Glycosylation defining cancer malignancy: new wine in an old bottle. Proc. Natl Acad. Sci. USA.

[8] Dwek RA (1995). Glycobiology: ‘towards understanding the function of sugars’. Biochem. Soc. Trans..

[9] Dennis JW, Granovsky M, Warren CE (1999). Protein glycosylation in development and disease. Bioessays.

[10] Miwa N, Uebi T, Kawamura S (2008). S100-annexin complexes-biology of conditional association. FEBS J..

[11] Hassan MS (2017). A novel intraperitoneal metastatic xenograft mouse model for survival outcome assessment of esophageal adenocarcinoma. PLoS ONE.

[12] Johnson GL, Lapadat R (2002). Mitogen-activated protein kinase pathways mediated by ERK, JNK, and p38 protein kinases. Science.

[13] Karnoub AE, Weinberg RA (2008). Ras oncogenes: split personalities. Nat. Rev. Mol. Cell Biol..

[14] Wellbrock C, Karasarides M, Marais R (2004). The RAF proteins take centre stage. Nat. Rev. Mol. Cell Biol..

[15] Yarden Y, Sliwkowski MX (2001). Untangling the ErbB signalling network. Nat. Rev. Mol. Cell Biol..

[16] Brown AP, Carlson TC, Loi CM, Graziano MJ (2007). Pharmacodynamic and toxicokinetic evaluation of the novel MEK inhibitor, PD0325901, in the rat following oral and intravenous administration. Cancer Chemother. Pharmacol..

[17] Hakomori S (1981). Glycosphingolipids in cellular interaction, differentiation, and oncogenesis. Annu. Rev. Biochem..

[18] Farooqui T, Franklin T, Pearl DK, Yates AJ (1997). Ganglioside GM1 enhances induction by nerve growth factor of a putative dimer of TrkA. J. Neurochem..

[19] Pryor S, McCaffrey G, Young LR, Grimes ML (2012). NGF causes TrkA to specifically attract microtubules to lipid rafts. PLoS ONE.

[20] Tsui-Pierchala BA, Encinas M, Milbrandt J, Johnson M (2002). Lipid rafts in neuronal signaling and function. Trends Neurosci..

[21] Prinetti A, Iwabuchi K, Hakomori S (1999). Glycosphingolipid-enriched signaling domain in mouse neuroblastoma Neuro2a cells. J. Biol. Chem..

[22] Ravindranath MH (2004). Gangliosides of organ-confined versus metastatic androgen-receptor-negative prostate cancer. Biochem. Biophys. Res. Commun..

[23] Bhuiyan RH (2016). Expression analysis of 0-series gangliosides in human cancer cell lines with monoclonal antibodies generated using knockout mice of ganglioside synthase genes. Glycobiology.

[24] Zarei M, Müthing J, Peter-Katalinić J, Bindila L (2020). Separation and identification of GM1b pathway Neu5Ac- and Neu5Gc gangliosides by on-line nanoHPLC-QToF MS and tandem MS: toward glycolipidomics screening of animal cell lines. Glycobiology.

[25] Kroes RA (2010). Overexpression of ST6GalNAcV, a ganglioside-specific alpha2,6-sialyltransferase, inhibits glioma growth in vivo. Proc. Natl Acad. Sci. USA.

[26] Anjum R, Blenis J (2008). The RSK family of kinases: emerging roles in cellular signalling. Nat. Rev. Mol. Cell Biol..

[27] Giese A, Bjerkvig R, Berens ME, Westphal M (2003). Cost of migration: invasion of malignant gliomas and implications for treatment. J. Clin. Oncol..

[28] Farin A (2006). Transplanted glioma cells migrate and proliferate on host brain vasculature: a dynamic analysis. Glia.

[29] Brenner MK (1997). Hematological malignancies. FASEB J..

[30] Armstrong DK (2006). Intraperitoneal cisplatin and paclitaxel in ovarian cancer. N. Engl. J. Med..

[31] Ye J (2016). Improved safety and efficacy of a lipid emulsion loaded with a paclitaxel-cholesterol complex for the treatment of breast tumors. Oncol. Rep..

[32] Tarr BD, Sambandan TG, Yalkowsky SH (1987). A new parenteral emulsion for the administration of taxol. Pharm. Res..

[33] Hansen MG, Nielsen SE, Berg K (1989). Re-examination and further development of a precise and rapid dye method for measuring cell growth/cell kill. J. Immunol. Methods..

