# Supplementary material for: Dicalcin suppresses invasion and metastasis of mammalian ovarian cancer cells by regulating the ganglioside-Erk1/2 axis
Source: Commun Biol. 2023 Oct 6;6:1015. doi: 10.1038/s42003-023-05324-w (PMC10558574; doi:10.1038/s42003-023-05324-w)
Supplement: Supplementary file 1 — Supplementary Information [file 42003_2023_5324_MOESM1_ESM.pdf]

Supplementary information for

Dicalcin suppresses invasion and metastasis of mammalian ovarian cancer cells by regulating the ganglioside-Erk1/2 axis.

Naofumi Miwa[1], Mayu Hanaue[1], Kayo Aoba[1], Ryohei Saito[1] and Ken Takamatsu[2]
[1]Department of Physiology, School of Medicine, Saitama Medical Unversity, Moro-hongo 38, Moroyama, Iruma-gun, Saitama 350-0495, Japan. [2]Department of Physiology, School of Medicine, Toho University, 5-21-16 Omori-nishi, Ohta-ku, Tokyo 143-8540, Japan.

Supplementary Fig. 1 Absence of endogenous dicalcin in OV2944 cells.
OV2944 cells were treated with anti-dicalcin antibody (red). Anti-dicalcin antibody showed no immunoreactivity with OV2944 cells, indicating the lack of endogeneous dicalcin inside the cells. Scale: 10 μm.

Supplementary Fig. 2 Binding of exogenously administered human dicalcin to OVCAR-3 cells and effect of human dicalcin treatment on the cell viability of OVCAR-3 cells.
(**a**) Left, Representative confocal image of OVCAR-3 cells treated with TMR-labeled human dicalcin (hDC) in the presence of Ca^{2+}; Right; Representative confocal image of OVCAR-3 cells treated with TMR-labeled hDC in the presence of 3mM EGTA; Scale: 0 μm. TMR-labeled hDC bound to OVCAR-3 cells in the presence of Ca^{2+}, but not in the absence of Ca^{2+}. Scale 10μm.
(**b**) Effect of human dicalcin on the cell viability of OVCAR-3 cells. OVCAR-3, human ovarian tumor cells were treated with human dicalcin (hDC) or BSA (10 μM each), and analyzed by MTT assay. The fluorescent data (OD_{560}) was normalized and evaluated. The graph shows mean data (n=6-9, mean±s.e.m). Circles show individual data. The result showed that dicalcin did not affect the cell viability of OVCAR-3 cells.

Supplementary Fig. 3 Sequence of mouse dicalcin and synthetic peptides.
The sequence of mouse dicalcin was divided into seven regions (P1-P7), considering the preservation of the native α-helix structure to the extent possible.

Supplementary Fig. 4 Binding of the synthetic peptide (P1) to OV2944 cells.
Upper: The sequence of mouse dicalcin was divided into seven regions (P1-P7). Rhodamine-labeled peptides corresponding to each region were probed on OV2944 cells. Representative confocal image of OV2944 cells treated with rhodamine-labeled P1 (Rhod-P1), DAPI (DAPI). BF, bright field image. Scale 50μm. Lower; Images of OV2944 cells at a higher magnification. Scale 10μm.

Supplementary Fig. 5 Examination of binding of the synthetic peptide (P2) to OV2944 cells. Representative confocal image of OV2944 cells treated with rhodamine-labeled P2 (Rhod-P2), DAPI (DAPI). BF, bright field image. Scale 10μm.

Supplementary Fig. 6 Examination of binding of the synthetic peptide (P3) to OV2944 cells. Representative confocal image of OV2944 cells treated with rhodamine-labeled P3 (Rhod-P3), DAPI (DAPI). BF, bright field image. Scale 10μm.

Supplementary Fig. 7 Examination of binding of the synthetic peptide (P4) to OV2944 cells. Representative confocal image of OV2944 cells treated with rhodamine-labeled P4 (Rhod-P4), DAPI (DAPI). BF, bright field image. Scale 10μm.

Supplementary Fig. 8 Binding of the synthetic peptide (P5) to OV2944 cells.
Upper; Representative confocal image of OV2944 cells treated with rhodamine-labeled P5 (Rhod-P5), DAPI (DAPI). BF, bright field image. Scale 50μm. Lower; Higher-magnified images of OV2944 cells. Scale 10μm.

Supplementary Fig. 9 Binding of the synthetic peptide (P6) to OV2944 cells.
Upper; Representative confocal image of OV2944 cells treated with rhodamine-labeled P6 (Rhod-P6), DAPI (DAPI). BF, bright field image. Lower; Images of OV2944 cells at a higher manification. Scale 10μm.

Supplementary Fig. 10 Binding of the synthetic peptide (P7) to OV2944 cells.
Upper; Representative confocal image of OV2944 cells treated with rhodamine-labeled P7 (Rhod-P1), DAPI (DAPI). BF, bright field image. Lower; Images of OV2944 cells at

a higher manification. Scale 10μm.

Supplementary Fig. 11 Mapping of the amino acid region of dicalcin for its suppressive action on metastasis.

Location of the peptide (residues 69-79, highlighted and indicated by the arrows) that inhibited metastasis were mapped by spacefill (upper) and ribbon (lower) models of mouse dicalcin. This region was simulated to form α-helix with the short loop and represented on its molecular surface (Ribbon model).

Supplementary Fig. 12 Alignment of the amino acid sequences of human and mouse dicalcin and binding of human dicalcin-derived peptide to OVCAR-3 cells.

(**a**) Alignment of the amino acid sequences of human and mouse dicalcin and the amino acid sequences that correspond to P6 in human and mouse dicalcin.

(**b**) Binding of human dicalcin-derived peptide to OVCAR-3 cells.

Upper; Representative confocal images of OVCAR-3 cells treated with rhodamine-labeled human P6 (Rhod-hDC-P6), DAPI (DAPI). BF, bright field image. Lower; Representative confocal images of OVCAR-3 cells treated with rhodamine-labeled control peptide (Rhod-hDC-P2), DAPI (DAPI). BF, bright field image. Scale: 20 μm.

(**c**) Effect of human dicalcin-derived peptide on *in vitro* invasivity of OVCAR-3 cells. Pretreated OVCAR-3 cells either with human dicalcin-derived peptide (P6) or BSA were placed on the Matrigel-coated inserts, which was immersed into the well of a 24-well plate that contained DMEM with 10% FBS at 37 °C, 5% CO_2. After 16 h at 37 °C, the membrane on the upper insert was stripped and stained with crystal violet. The ratio of the number of stained cells/the number of spreader cells was normalized and evaluated as the index of invasion. The index for control was set to 100%. Numbers in the parentheses represent averaged values in each condition. The bar graph shows mean data (n=10-17, mean±s.e.m.). Circles show individual data. P values represent unpaired Student's *t*-test. Scale: 50 μm. Exogenously administered human dicalcin peptide (hDC-P6) suppressed *in vitro* invasion of OV2944 cells in a dose-dependent manner.

Supplementary Fig. 13 Inhibition of *in vitro* invasion of human prostate tumor cells

by P6.

(**a**) Binding of rhodamine-labelled P6 to PC-3 cells, human prostate cancer cells. Scale: 10 μm

(**b**) *In vitro* invasion assay. Exogenously administered P6 suppressed *in vitro* invasion of PC-3 cells. The graph shows mean data (mean±s.e.m., n=6, unpaired Student's *t*-test). Circles show individual data.

Supplementary Fig. 14 No effect on *in vitro* invasion of T-Ag-Mose cells by P6.

(a) Non-binding of rhodamine-labelled P6 to T-Ag-Mose cells, SV40-infected immortal mouse normal ovarian epithelial cellss. Scale: 10 μm

(b) *In vitro* invasion assay. Exogenously administered P6 did not affect *in vitro* invasion of T-Ag-Mose cells. The graph shows mean data (mean±s.e.m., n=8-10, unpaired Student's *t*-test). Circles show individual data.

Supplementary Fig. 15 Isolation of tdTomato-positive OV2944 cells by flow sorting and schedule of peptide injection.

OV2944 cells were transfected with tdTomato, and ~24 hs later, cells were analyzed to isolate tdTomato-positive cells using a flow cytomenter (FACS Aria). OV2944 tdTomato-transfected cells were pretreated either with P6 or P2 (as control), rinsed, and intraperitoneally injected into B6C3F1 mice. Peptide (P6 or P2, 3 nmoles/150 μL) was injected once per two days and thereafter until study end.

Supplementary Fig. 16 Effect of P6 administration on Erk1/2, p38 MAPK, and AKT in OVCAR3.

(a) Effect of P6 administration on Erk1/2 in OVCAR3. Upper: Cell extracts were prepared 30-min after the addition of P6 into the culture medium and a portion of them was subjected to western blot analysis. Lower: The ratio of pErk/total Erk for control was set to 100% and the data were normalized. The graph shows mean data (n=8-10, mean±s.e.m.). Circles show individual data. P values represent two-sided unpaired Student's *t*-test. (b) Effect of P6 administration on p38 MAPK in OVCAR3.

Cell extracts were prepared 30-min after the addition of P6 into the culture medium and a portion of them was subjected to western blot analysis. (c) Effect of P6 administration on AKT in OVCAR3. Upper: Cell extracts were prepared 30-min after the addition of

P6 into the culture medium and a portion of them was subjected to western blot analysis. Lower: The ratio of pErk/total Erk for control was set to 100% and the data were normalized. The graph shows mean data (n=4-6, mean±s.e.m.). Circles show individual data. P values represent two-sided unpaired Student's *t*-test.

Supplementary Fig. 17 P6 interacts with OV2944 cells mediated by its binding to GM1b.

(a) P6 binds to oligosaccharides of ganglioside. A glycan array of a variety of gangliosides was incubated with biotinylated P6. Following the treatment with Cy3-conjugated streptavidin, the fluorescent intensity was quantified. The graph shows mean data (n=3). (b) A schematic illustration of GM1b distribution in the lipid raft. Lipid raft internalizes transmembrane proteins, membrane-associated proteins, cholesterol as well as ganglioside. Gangliosides play multiple functions (*e.g.*, growth, differentiation, maturation) by modulating a variety of cellular signaling pathway. GM1b composes ceramide, glucose, galactose, N-acetyl galactosamine (GalNAc) and N-acetylneuraminic acid (Neu5Ac). It interacts with growth-factor receptor(s), influencing receptor-mediated signaling to exert cellular functions.

Supplementary Fig. 18 P6 binding to the cell membrane of OV2944 cells.

Representative confocal images of OV2944 cells treated with P6 and anti-CD44 antibody. OV2944 cells were treated with rhodamine-labeled P6 (Rhod-P6, red) and anti-CD44 antibody as a marker of the membrane (CD44, green). Scale: 10 μm. P6 bound to the cell membrane of OV2944 cells (arrows in merged image, yellow).

Supplementary Fig. 19 Loss of sensitivity to P6 by neuraminidase treatment of OV2944 cells.

OV2944 cells were treated with neuraminidase (NeuA) to eliminate the terminal sialic-acid of GM1b, followed by exogenous administration of rhodamine-p6 (Rhod-P6).

(a) Representative confocal images of OV2944 cells reacted with *Maackia amurensis* (MALII, red) and wheat germ agglutinin (WGA, green), following NeuA-pretreatment (+NeuA). Scale: 10 μm.

(b) Representative confocal images of OV2944 cells reacted with MALII (red) and WGA (green)(-NeuA). Scale: 10 μm.

(c,d) NeuA-pretreatment reduced the fluorescent signal of MLAII across the membrane of OV2944 cells. The site with the peak signal of WGA was considered the cell membrane (dashed line), and fluorescent signals of MALII and WGA were quantified across the membrane (approximately 1 μm width, white lines in a and b; n=20-30; mean±s.e.m.; a.u., optical arbitrary units). MALII signals were diminished after pretreatment of the cells with NeuA (+NeuA in c), confirming elimination of the terminal sialic-acid of GM1b. Please note that MLAII recognizes the terminal sialic-acid, whereas WGA does not.
(e) Representative confocal images of OV2944 cells reacted with rhodamine-labeled P6 (Rhod-P6), or anti-CD44 antibody as a marker of the cell membrane of OV2944 cells (CD44), following NeuA-pretreatment (+NeuA). Scale: 10 μm.
(f) Representative confocal images of OV2944 cells reacted with rhodamine-labeled P6 (Rhod-P6), or anti-CD44 antibody (CD44)(-NeuA). Scale: 10 μm.
(g,h) NeuA-pretreatment reduced the fluorescent signal of rhodamine-labeled P6 across the membarne of OV2944 cells. Fluorescent signals of rhodamine-lebeled P6 (Rhod-P6) and CD44 (CD44) were quantified across the membrane (approximately 1μm width, white lines in e and f; n=20-30, mean±s.e.m., a.u., optical arbitrary units). The fluorescent signals of rhodamine-labeled P6 (Rhod-P6) was decreased by treatment with NeuA (arrow in g). Note that the peak signal of CD44 indicates the site of the membrane of OV2944.

Supplementary Fig.20. Addition of PD0325901 cancelled the augmentation of Erk1/2 activity by GM1b administration to OV2944 cells.

Cells extracts were prepared 30-min after the addition of either GM1b alone (GM1b) or combined administration GM1b and PD0325901 (GM1b+PD0325901) into the medium and a portion of them was subjected to western blot analysis using phopho-Erk1/2 and total-Erk1/2 antibodies. The ratio of pErk/tErk for control was set to 100% and the data were normalized. The graph shows mean data (n=3-6, mean±s.e.m.). Circles show individual data. P-values represent two-sided unpaired Student's *t*-test.

Supplementary Fig. 21 A schematic model of suppressive action of dicalcin on metastasis through its binding to GM1b on cancer cells.

Dicalcin binds to GM1b in the lipid raft of cancer cell membrane, hampering proper

interaction of GM1b and receptor(s), downregulating Erk1/2 activity to suppress migratory activity of cancer cells.

Supplementary Fig. 22 Unedited/uncropped western blot gels for Fig.1f.

Supplementary Fig. 23 Unedited/uncropped western blot gels for Fig.3b.

Supplementary Fig. 24 Unedited/uncropped western blot gels for Fig.3d.

Supplementary Fig. 25 Unedited/uncropped western blot gels for Fig.4f.

Supplementary Fig. 26 Unedited/uncropped western blot gels for Supplementary Fig.16.

Supplementary Fig. 27 Unedited/uncropped western blot gels for Supplementary Fig.20.

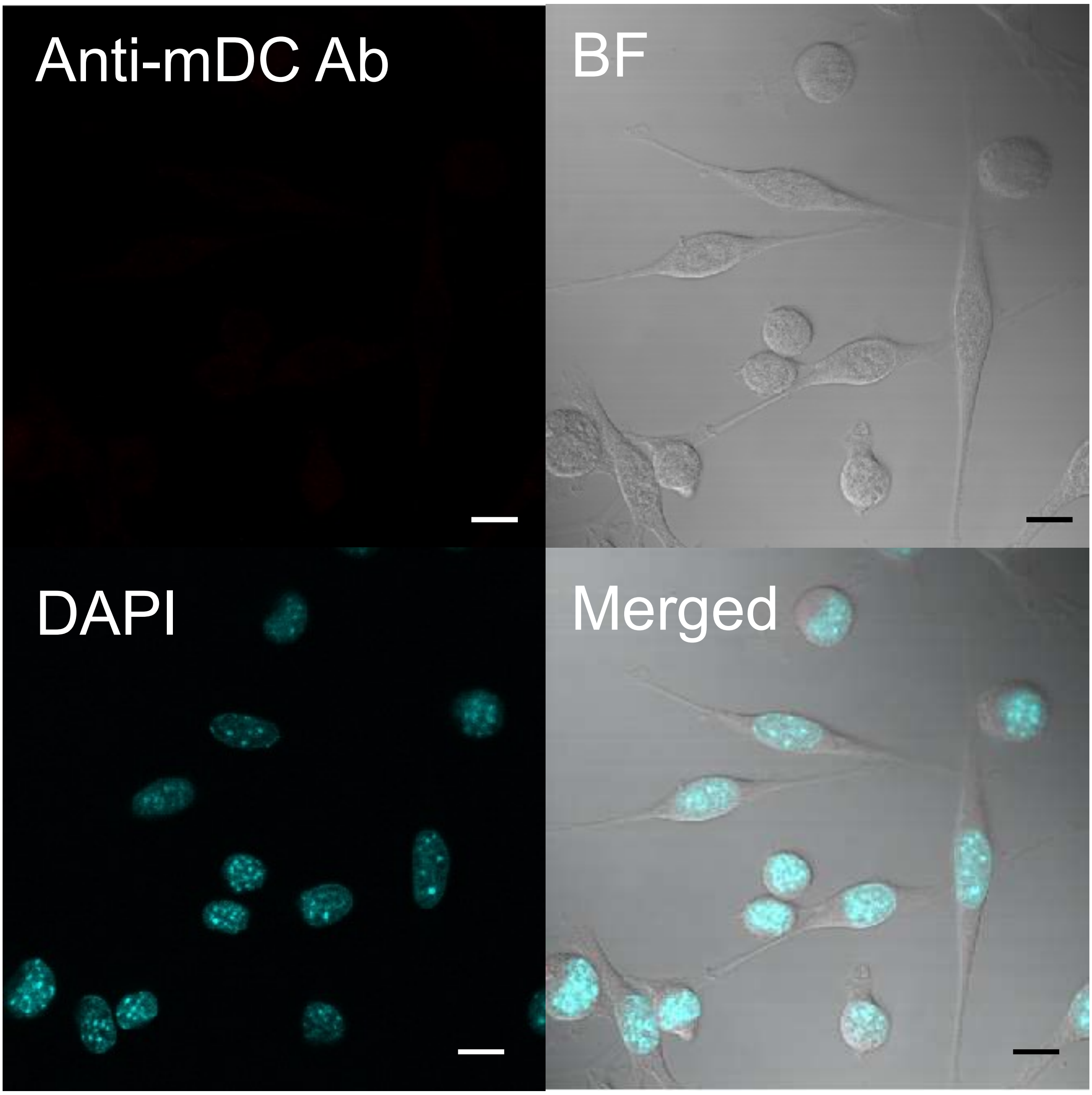

Supplementary Fig.1

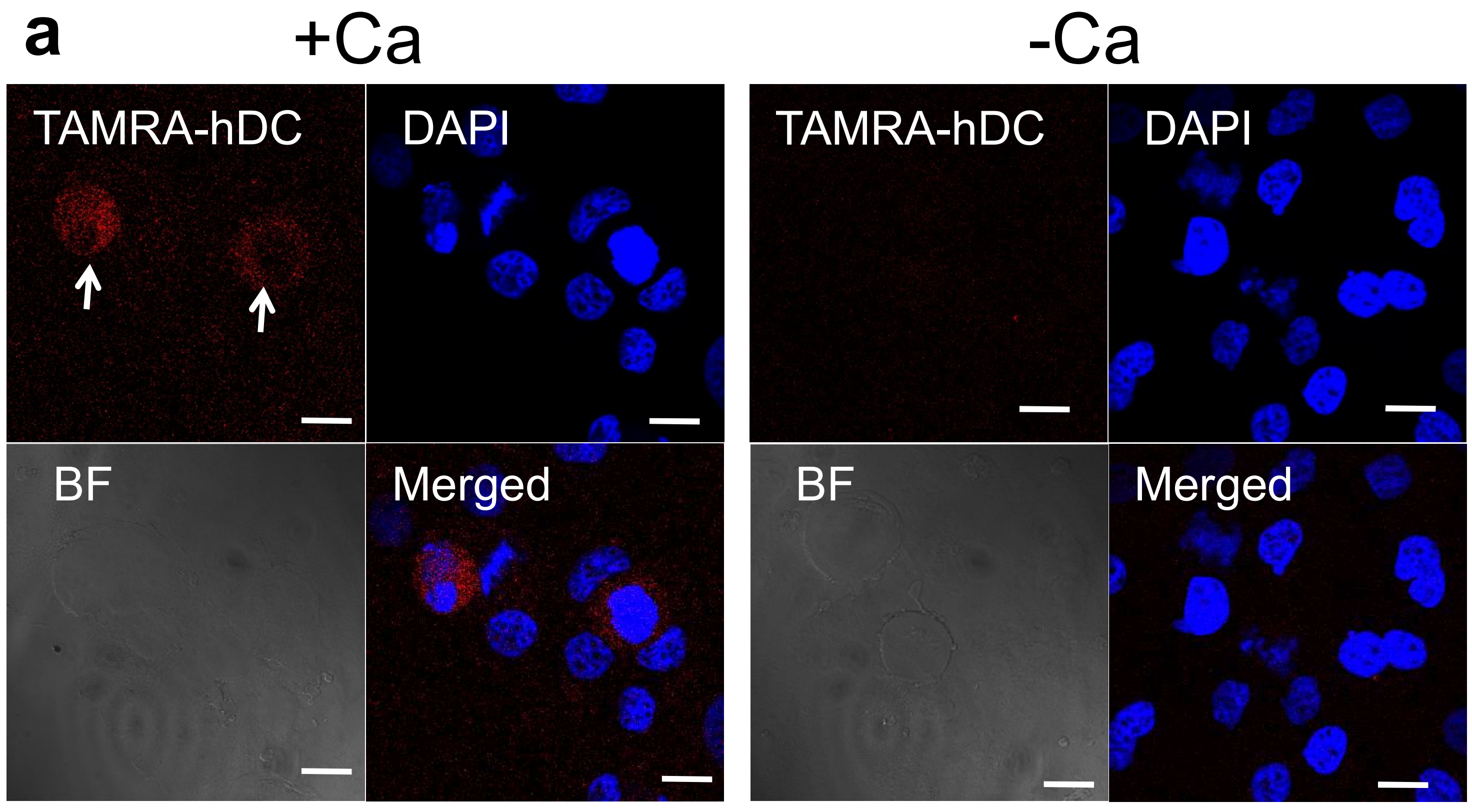

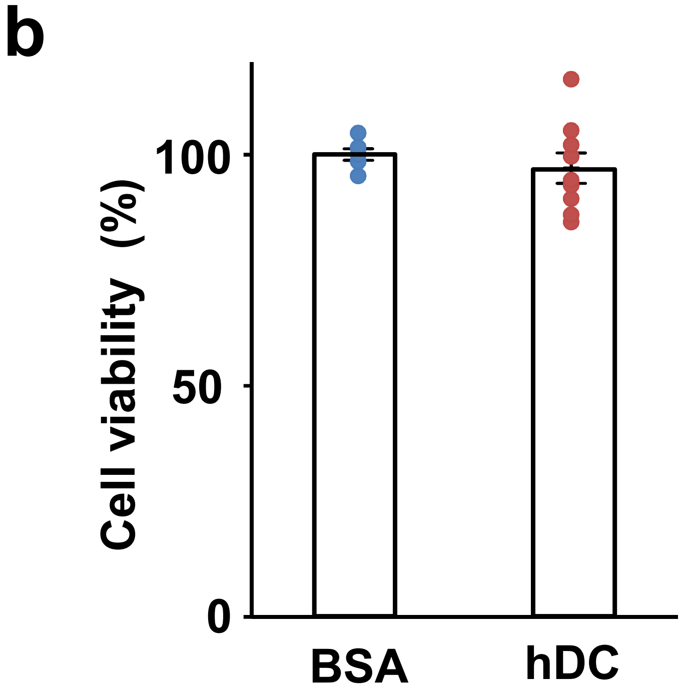

Supplementary Fig.2

Sequence of mouse dicalcin and synthetic peptides

1 PTETERCIESLIAVFQKYSGKDGNNTQLSKTEFLSF 36

P1 P2 P3

37 MNTELAAFTKNQKDPGVLDRMMKKLDLNCDG 67

P4 P5

68 QLDFQEFLNLIGGLAIACHDSFIQTSQKRI 97

P6 P7

P1
PTETERCIE

P2
SLIAVFQKY

P3
SGKDGNNTQLSKTEFLSF

P4
MNTELAAFTKNQKDPGVLDR

P5
MMKKLDLNCDG

P6
QLDFQEFLNLI

P7
GGLAIACHDSFIQTSQKRI

Supplementary Fig.3

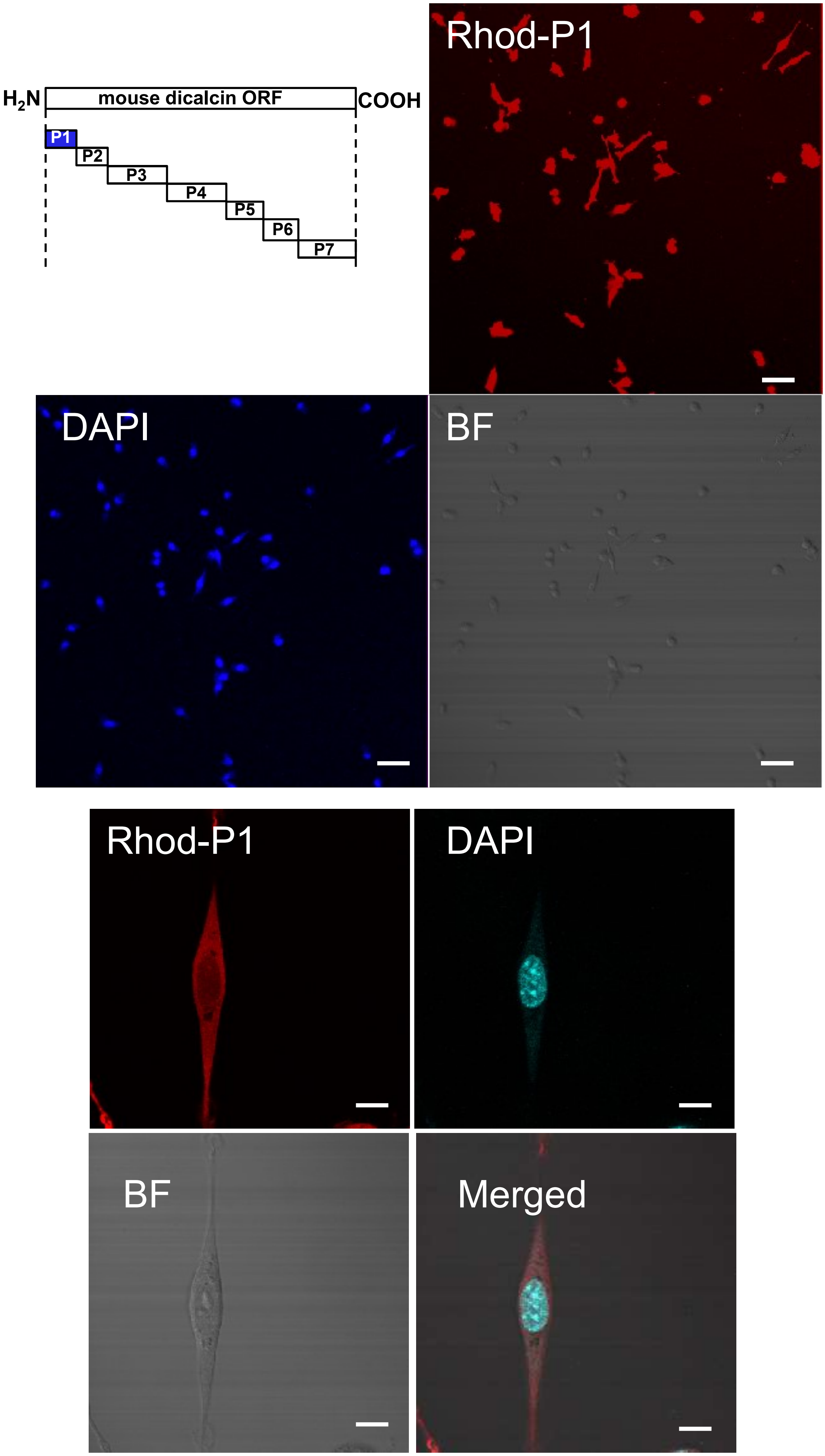

Supplementary Fig.4

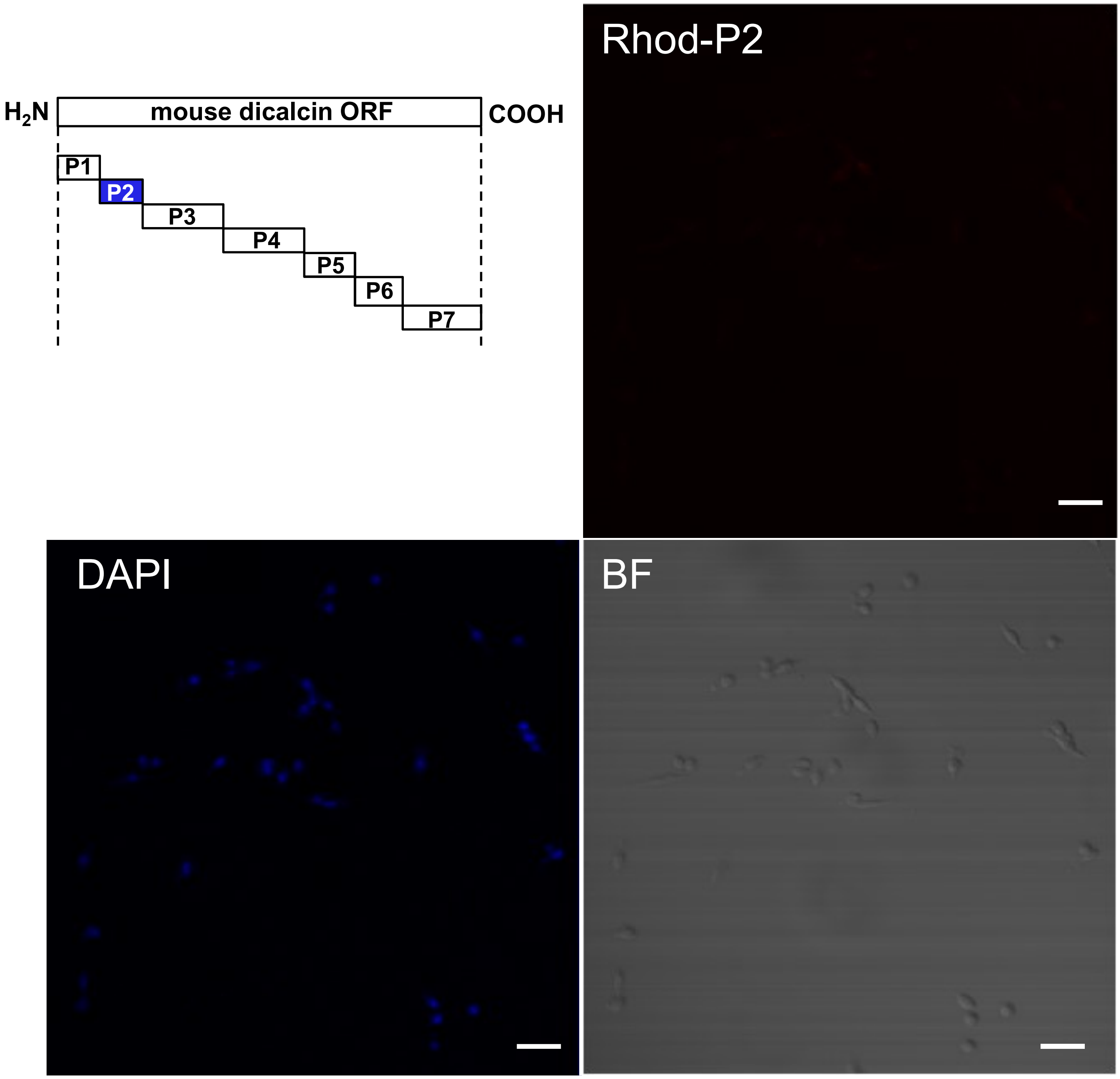

SupplementaryFig.5

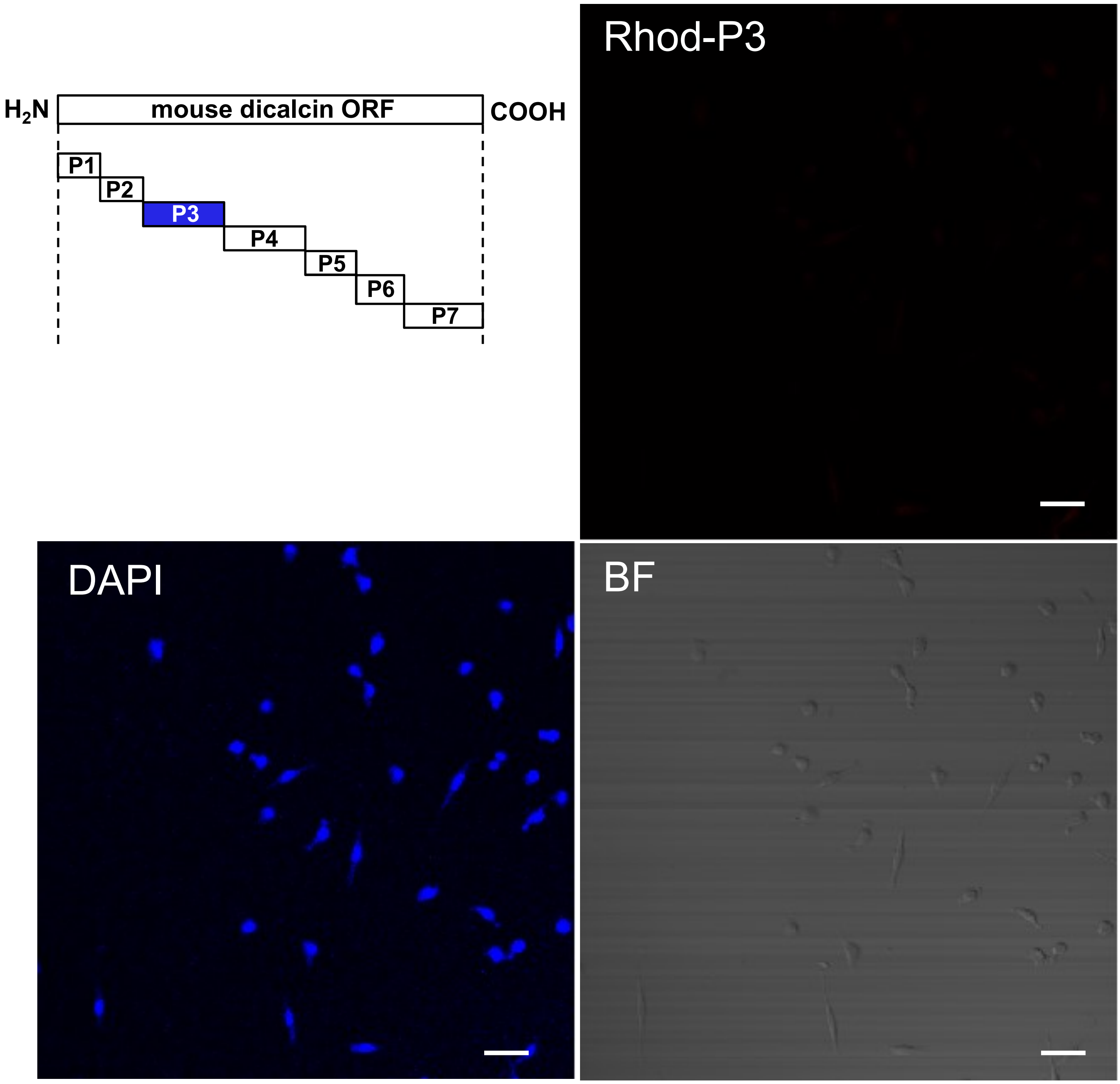

SupplementaryFig.6

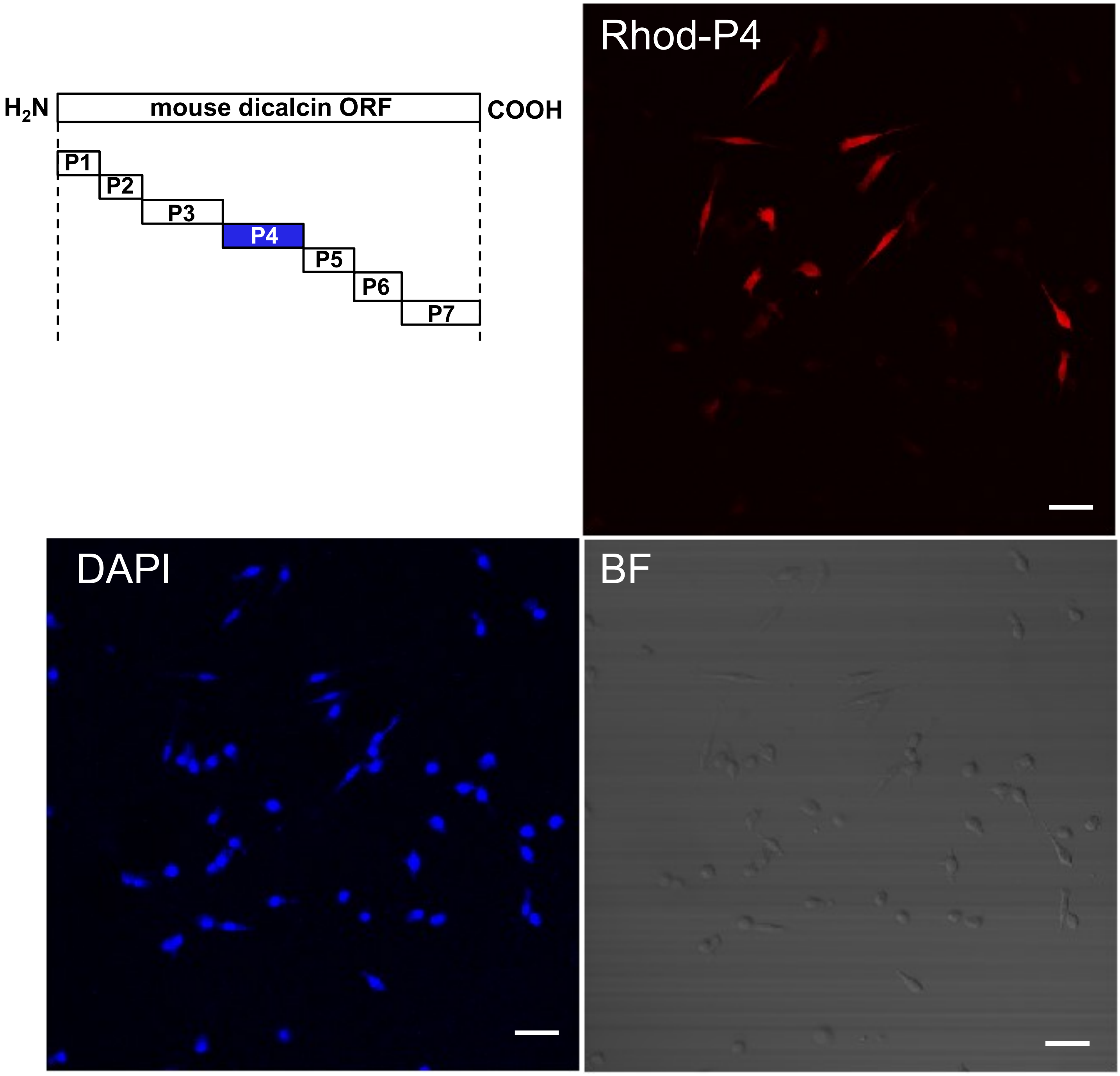

Supplementary Fig.7

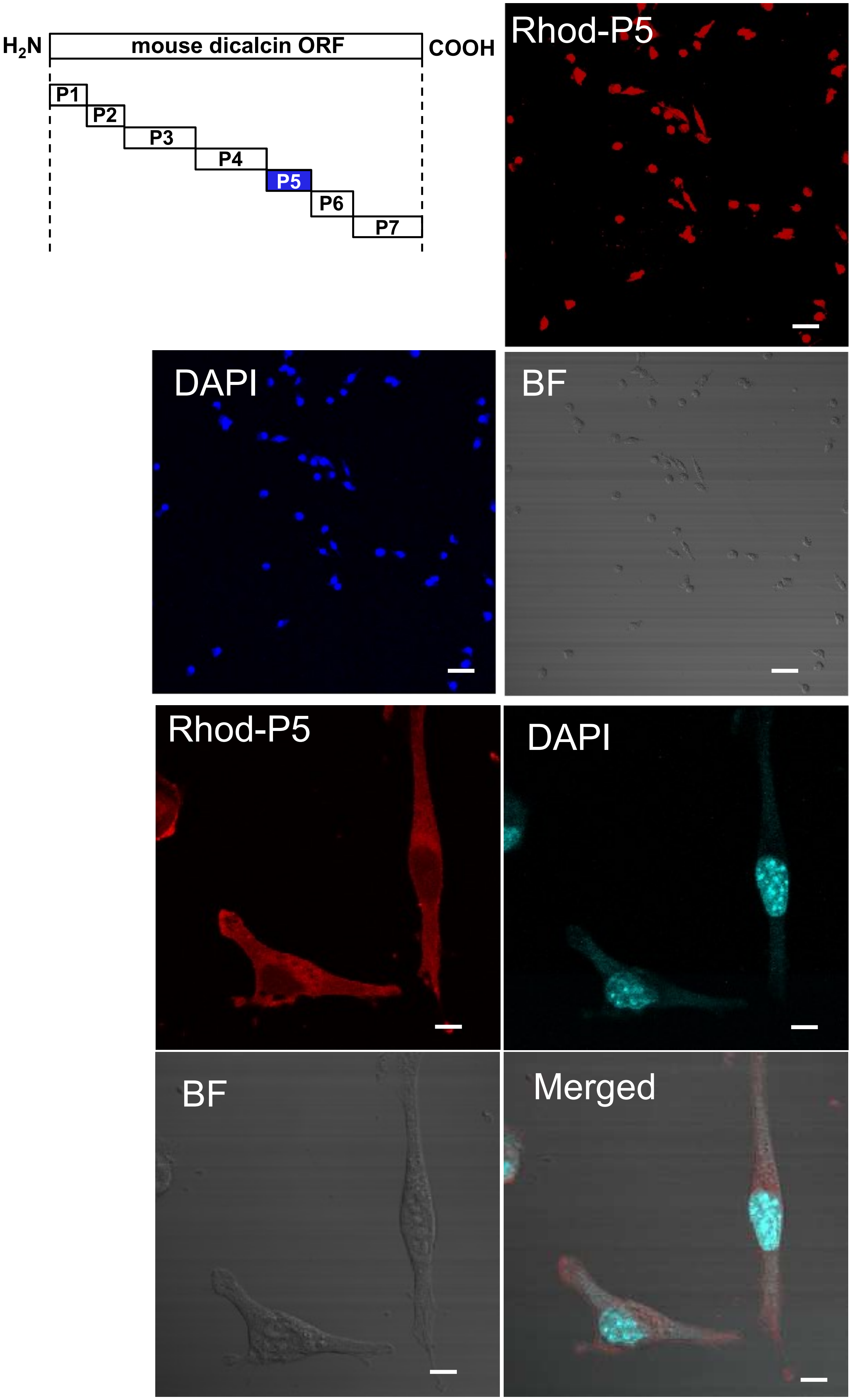

Supplementary Fig.8

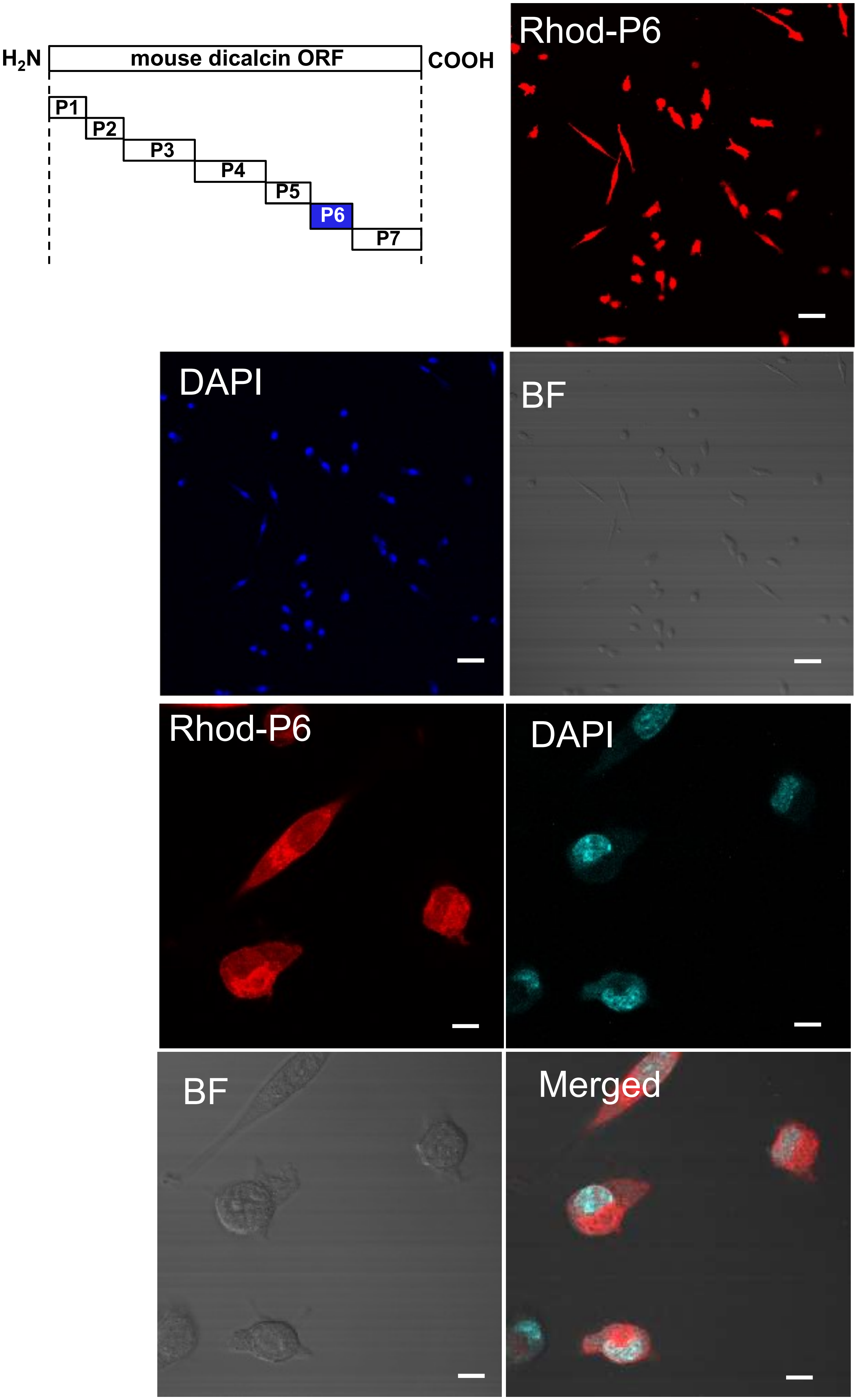

Supplementary Fig.9

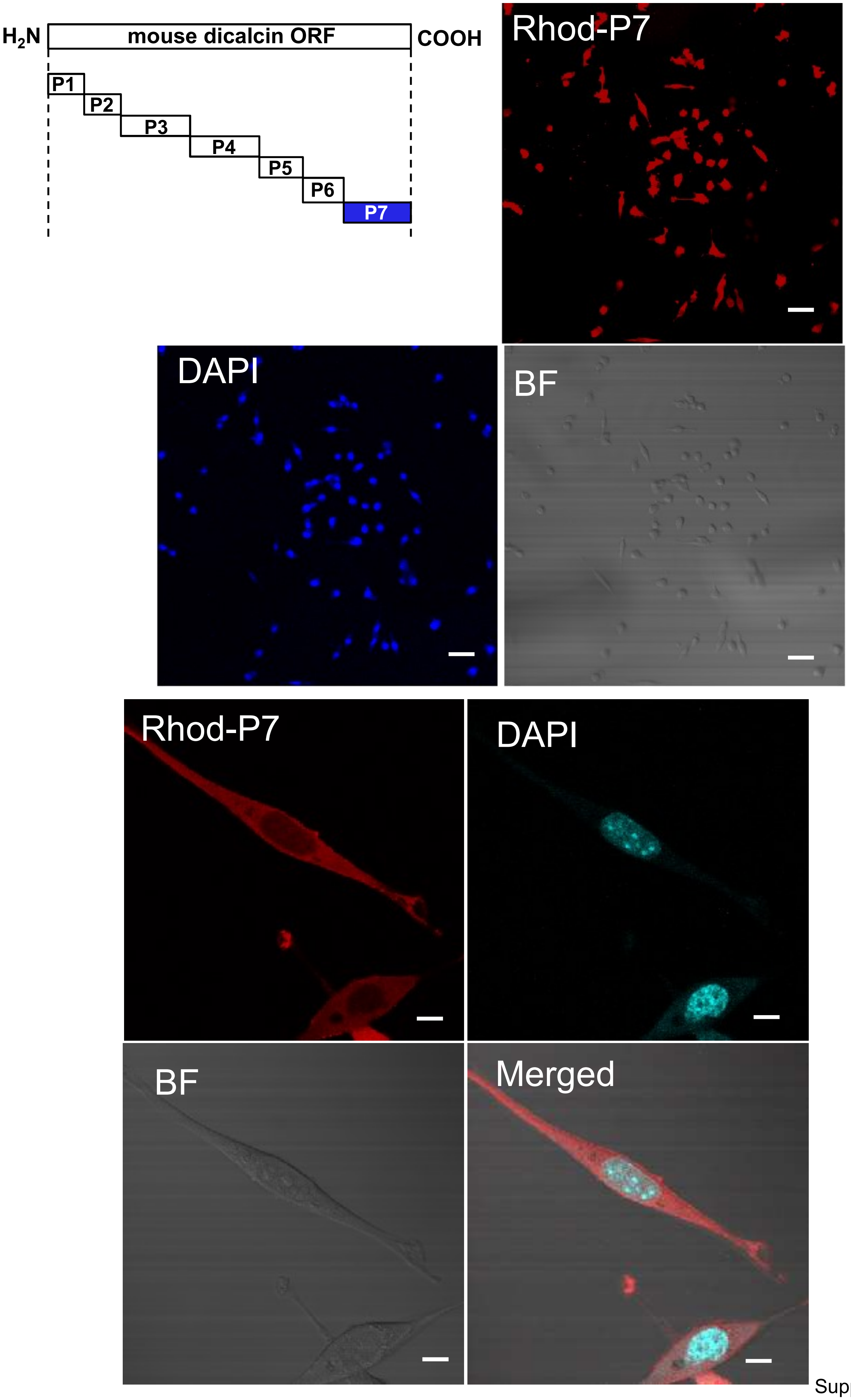

Supplementary Fig.10

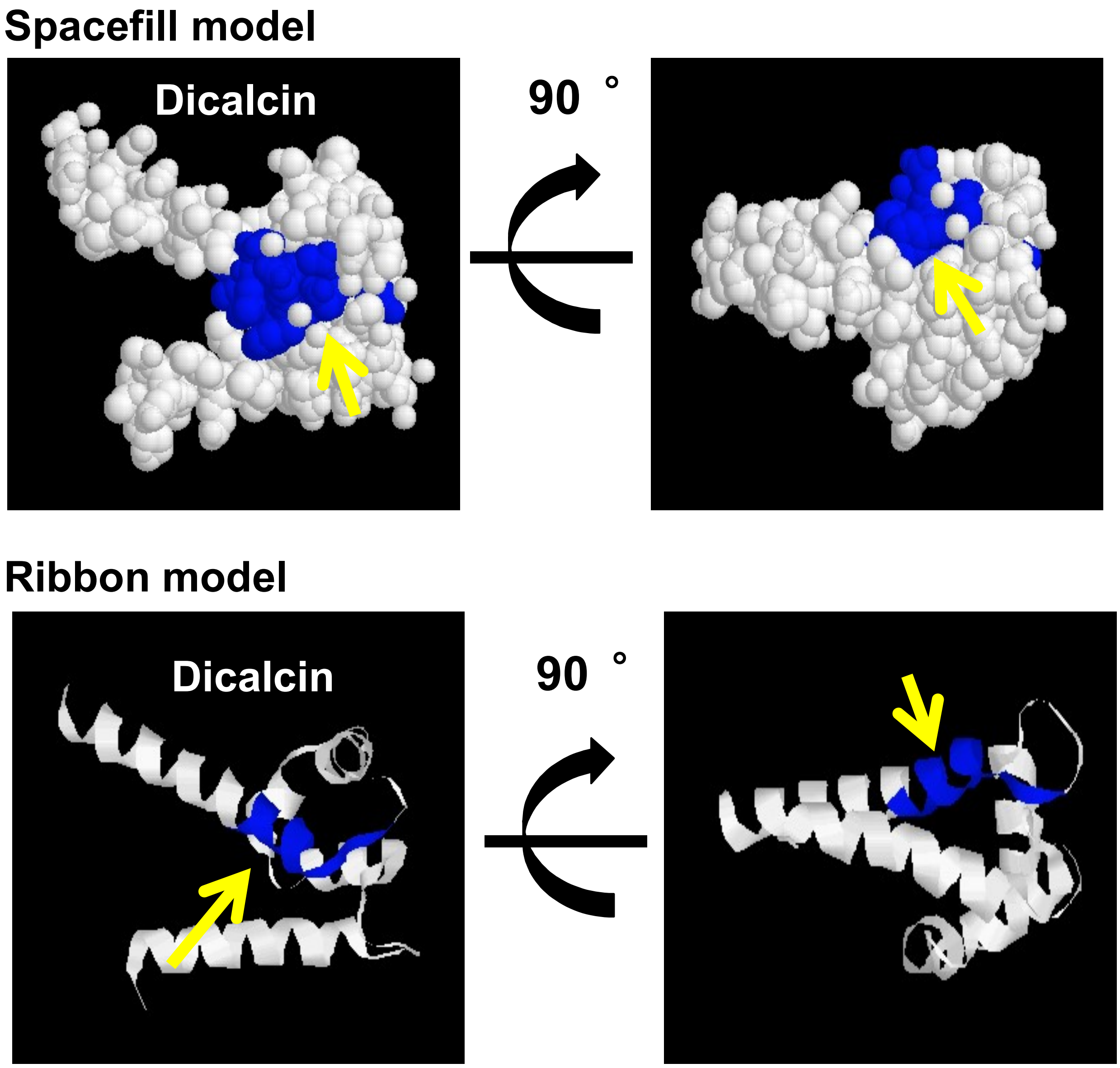

Supplementary Fig.11

a

```
Human   7    PTETERCIESLIAVFQKYAGKDGYNYTLSKTEFLSFMNTELAAFTKNQKDPGVLDRMMKK   66
             PTETERCIESLIAVFQKY+GKDG  N   LSKTEFLSFMNTELAAFTKNQKDPGVLDRMMKK
Mouse   2    PTETERCIESLIAVFQKYSGKDGNNTQLSKTEFLSFMNTELAAFTKNQKDPGVLDRMMKK   61

Human  67    LDTNSDGQLDFSEFLNLIGGLAMACHDSFLKAVPSQKR   104
             LD N  DGQLDF  EFLNLIGGLA+ACHDSF++     SQKR
Mouse  62    LDLNCDGQLDFQEFLNLIGGLA I ACHDSFIQT--   SQKR    97
```

Mouse P6: QLDFSEFLNLI

Human P6: QLDFQEFLNLI

11 amino acids

Identiy = 90% (10/11)

b

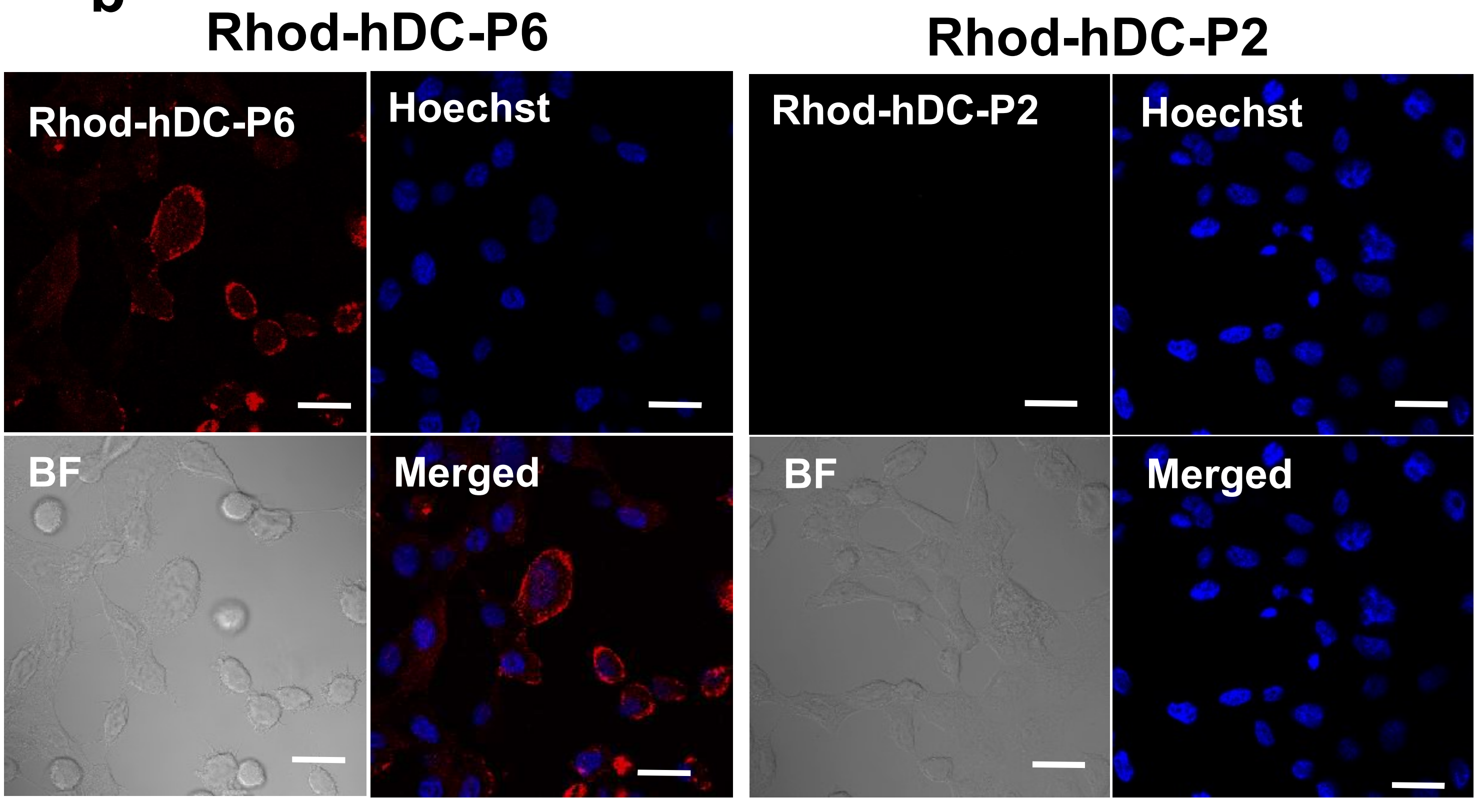

C

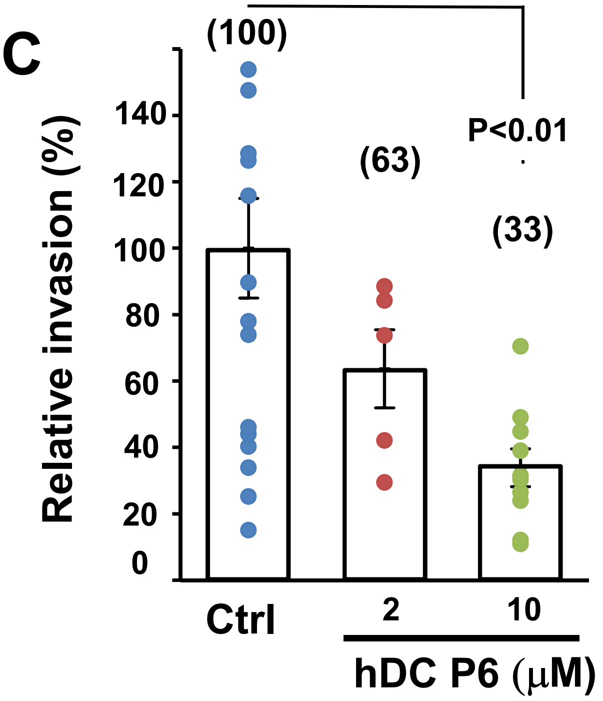

Supplementary Fig.12

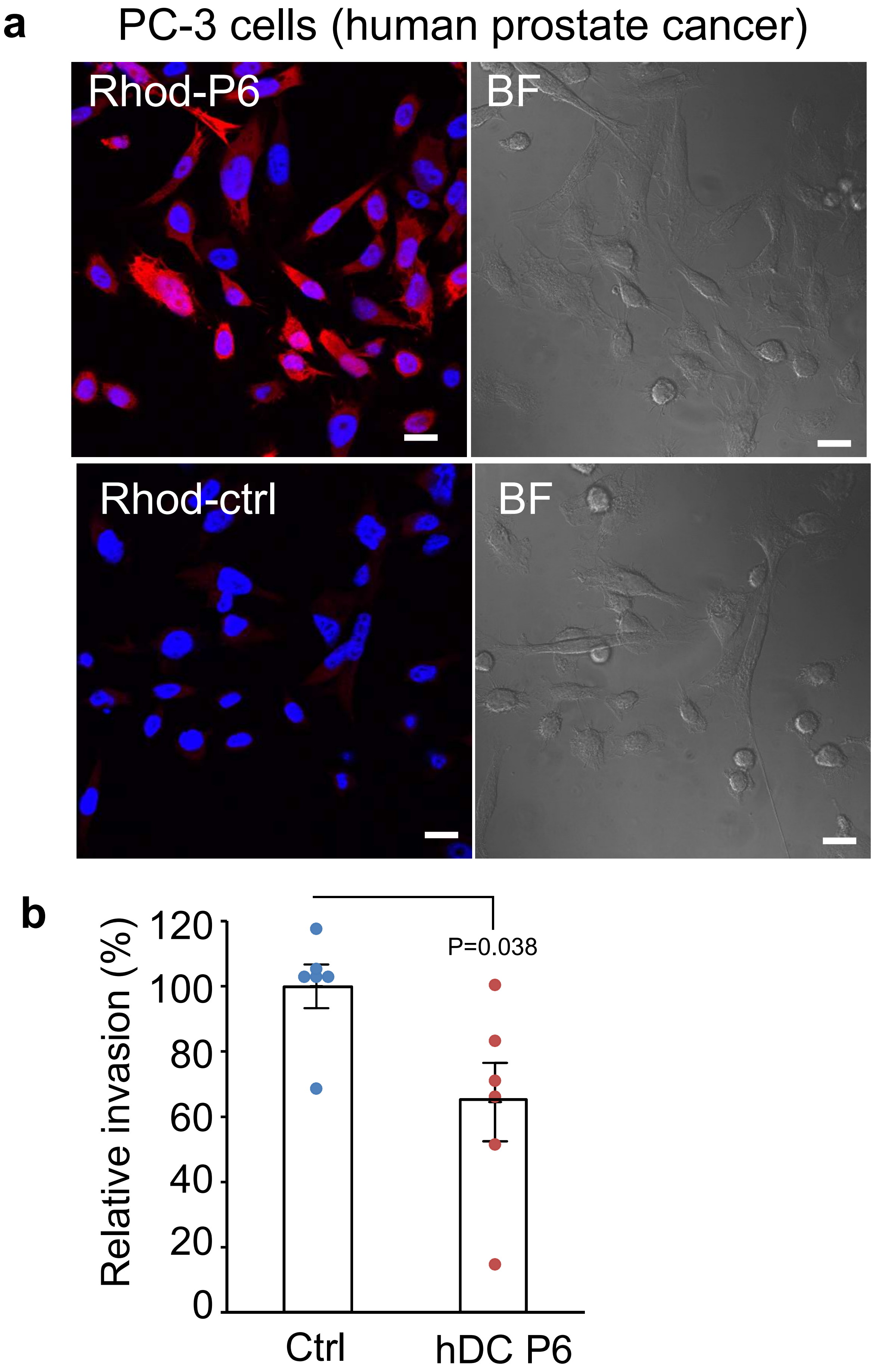

Supplementary Fig.13

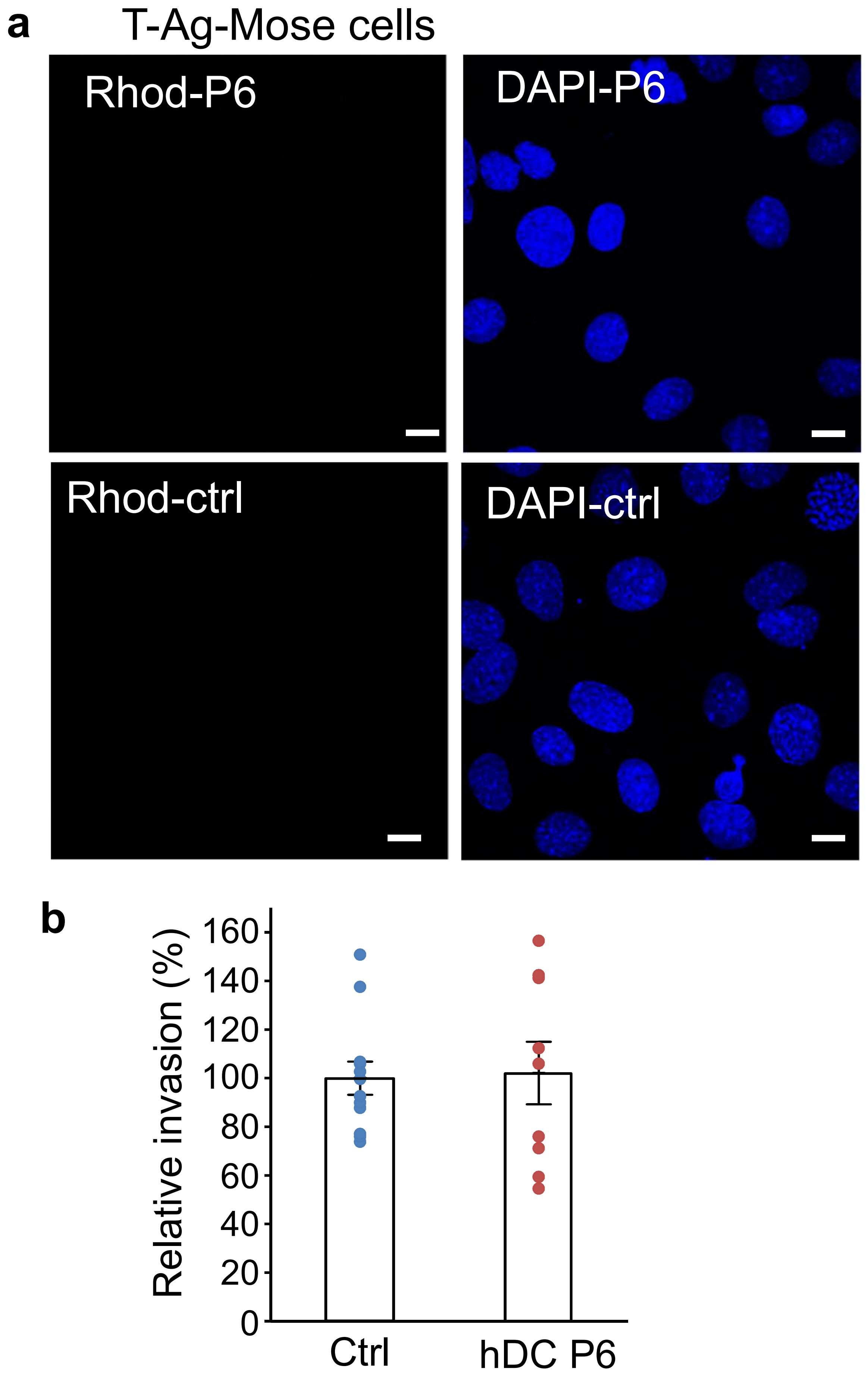

Supplementary Fig.14

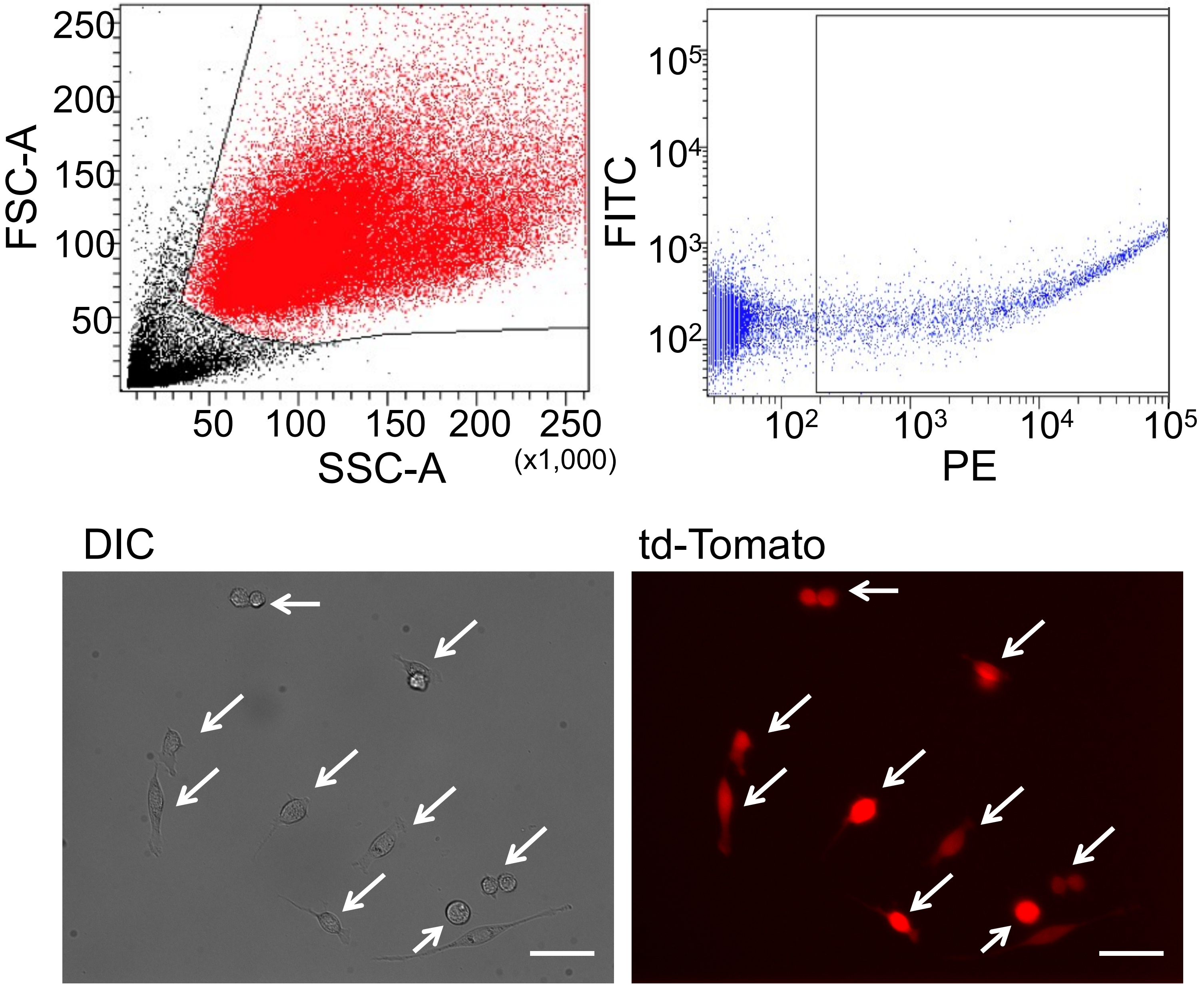

Schedule of the intraperitoneal injection

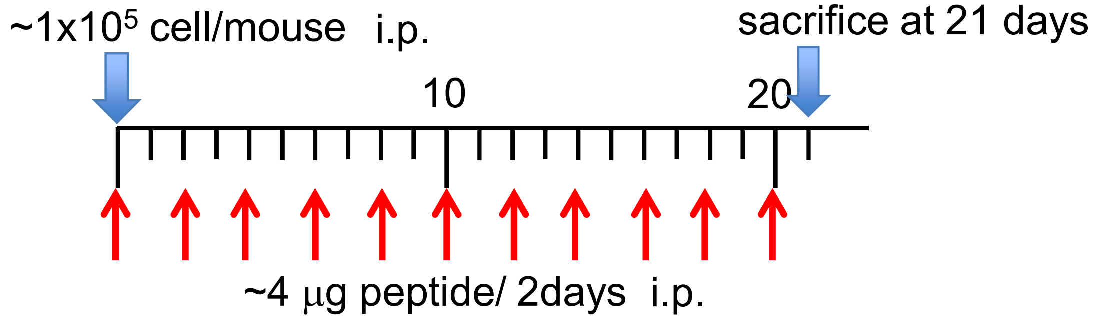

Supplementary Fig.15

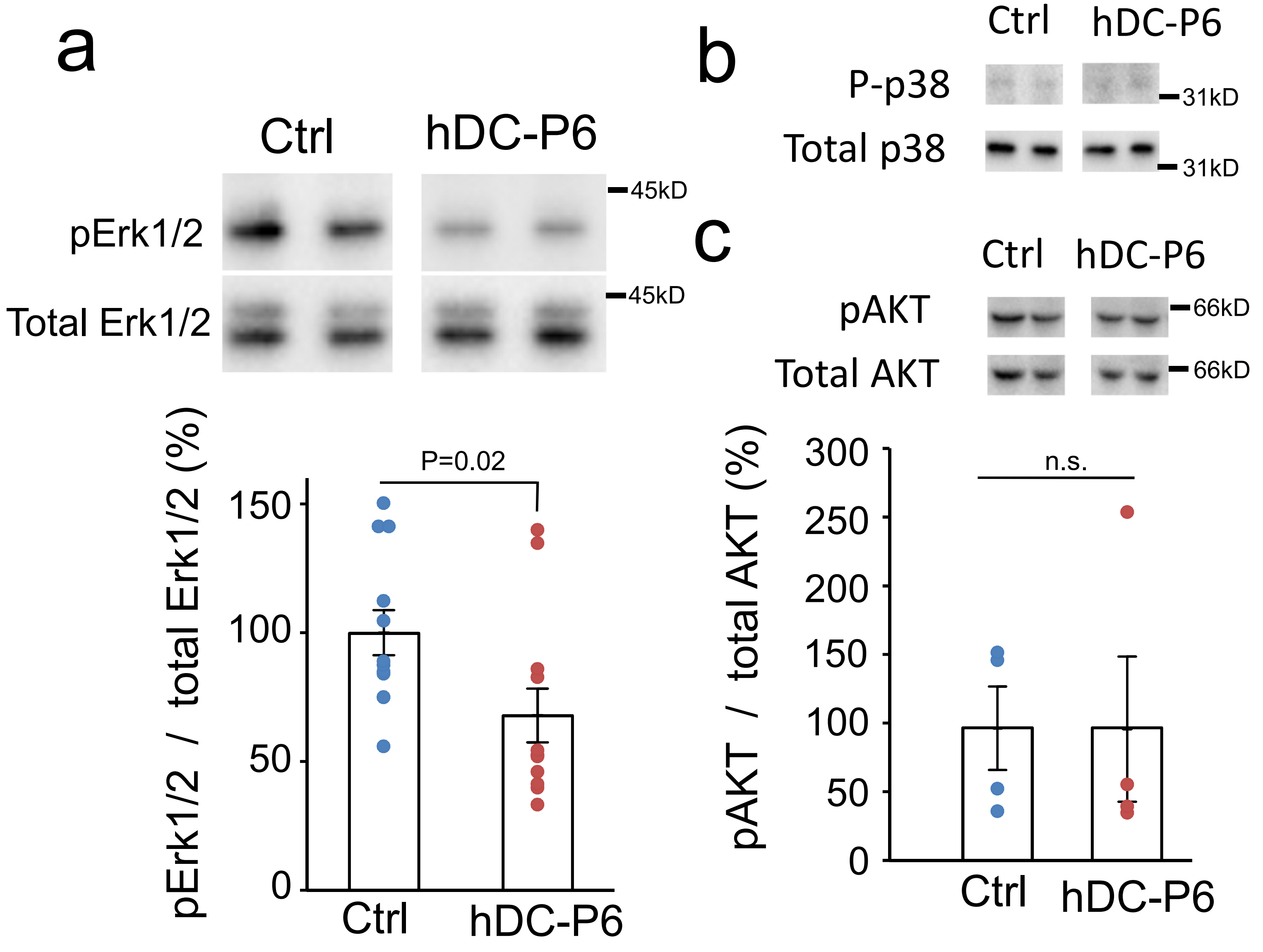

Supplementary Fig.16

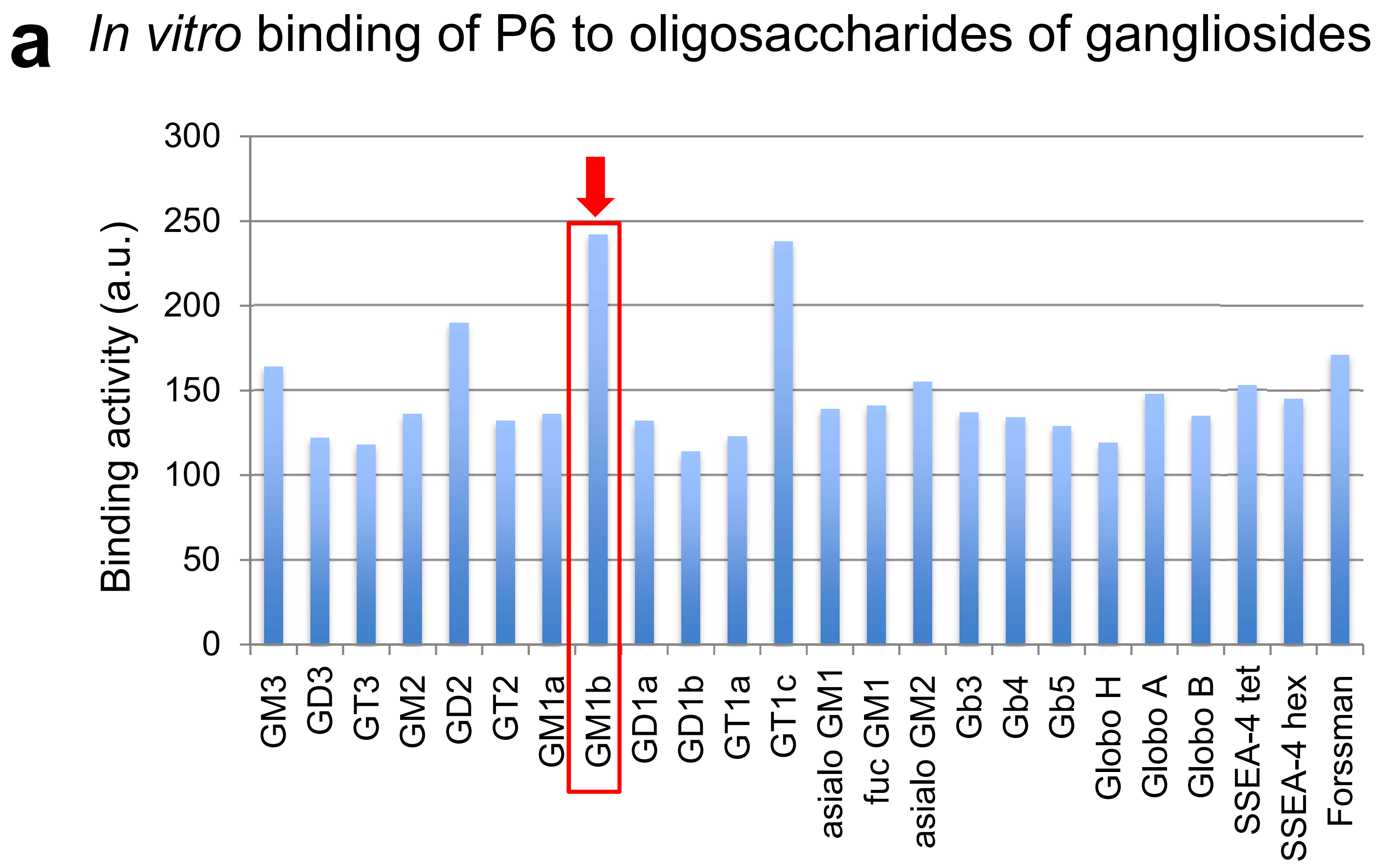

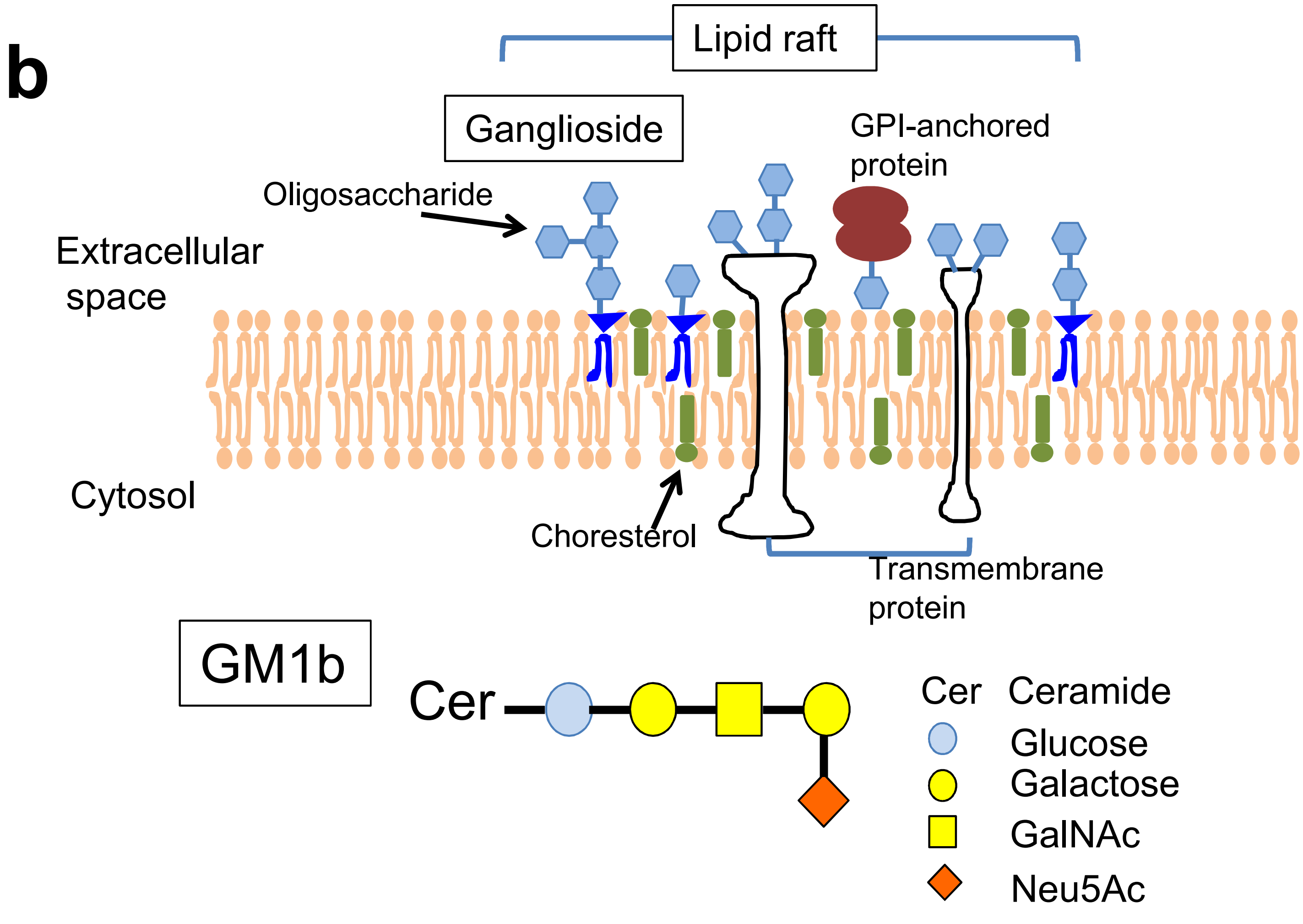

Supplementary Fig.17

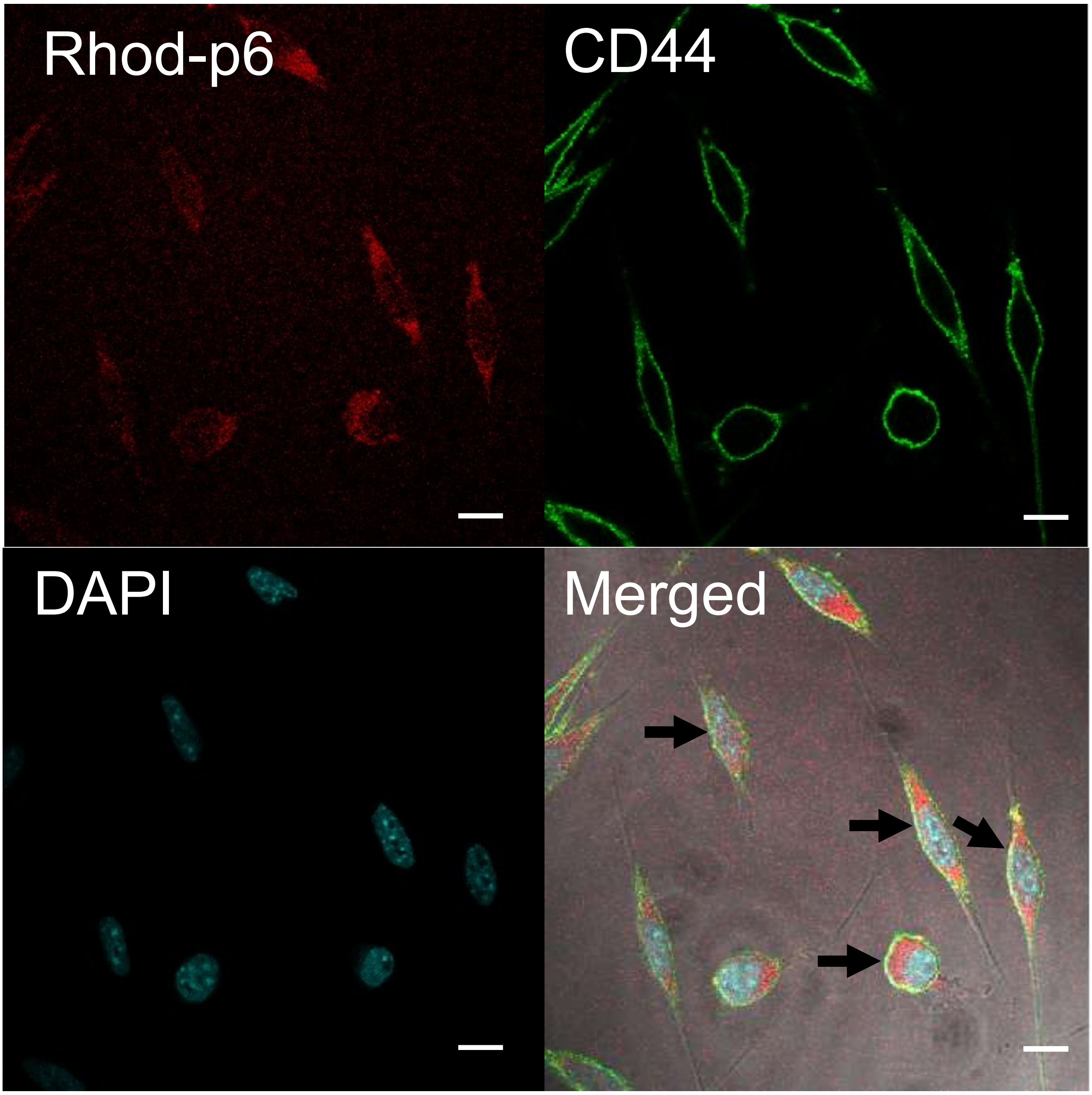

Supplementary Fig. 18

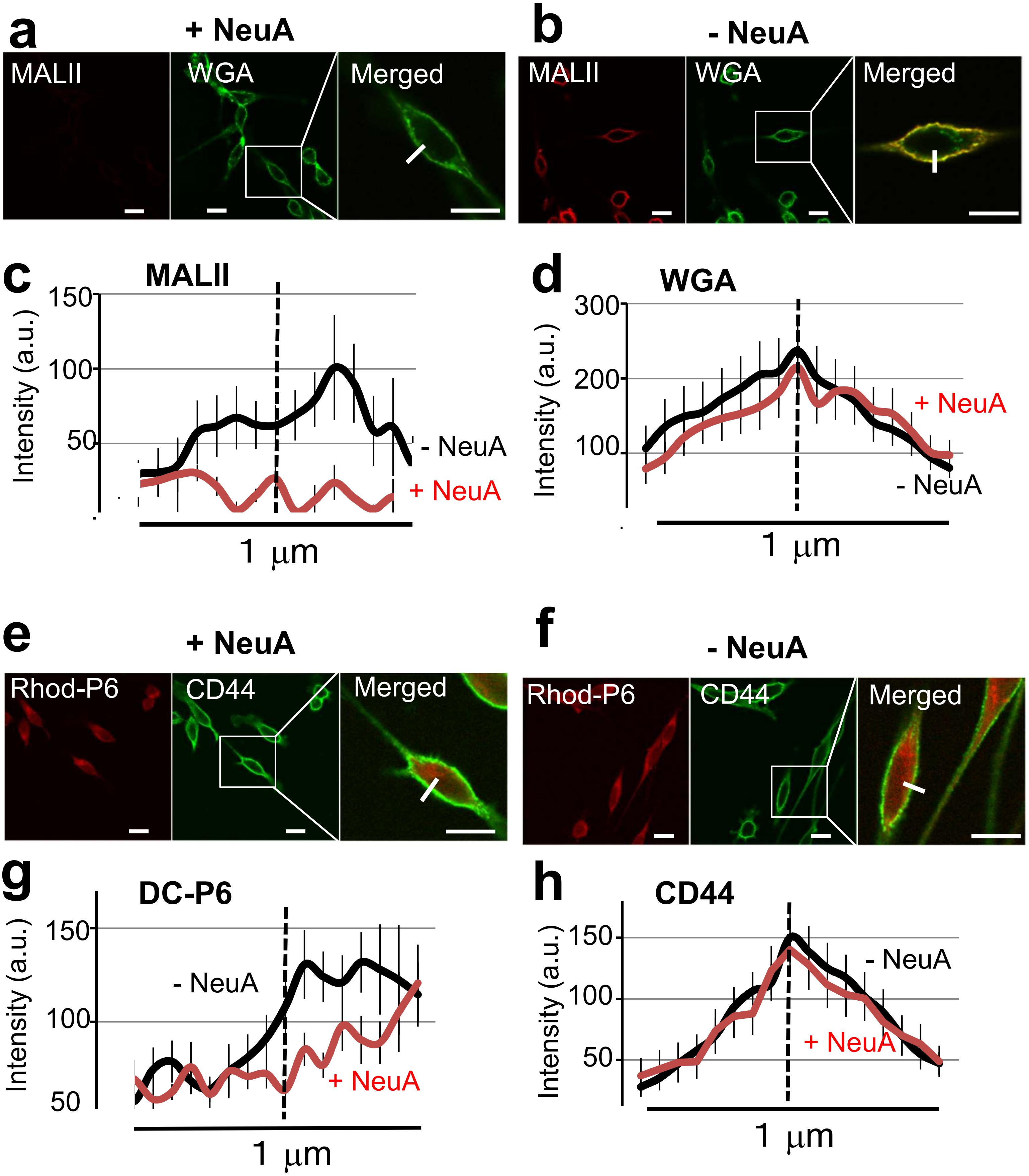

Supplementary Fig. 19

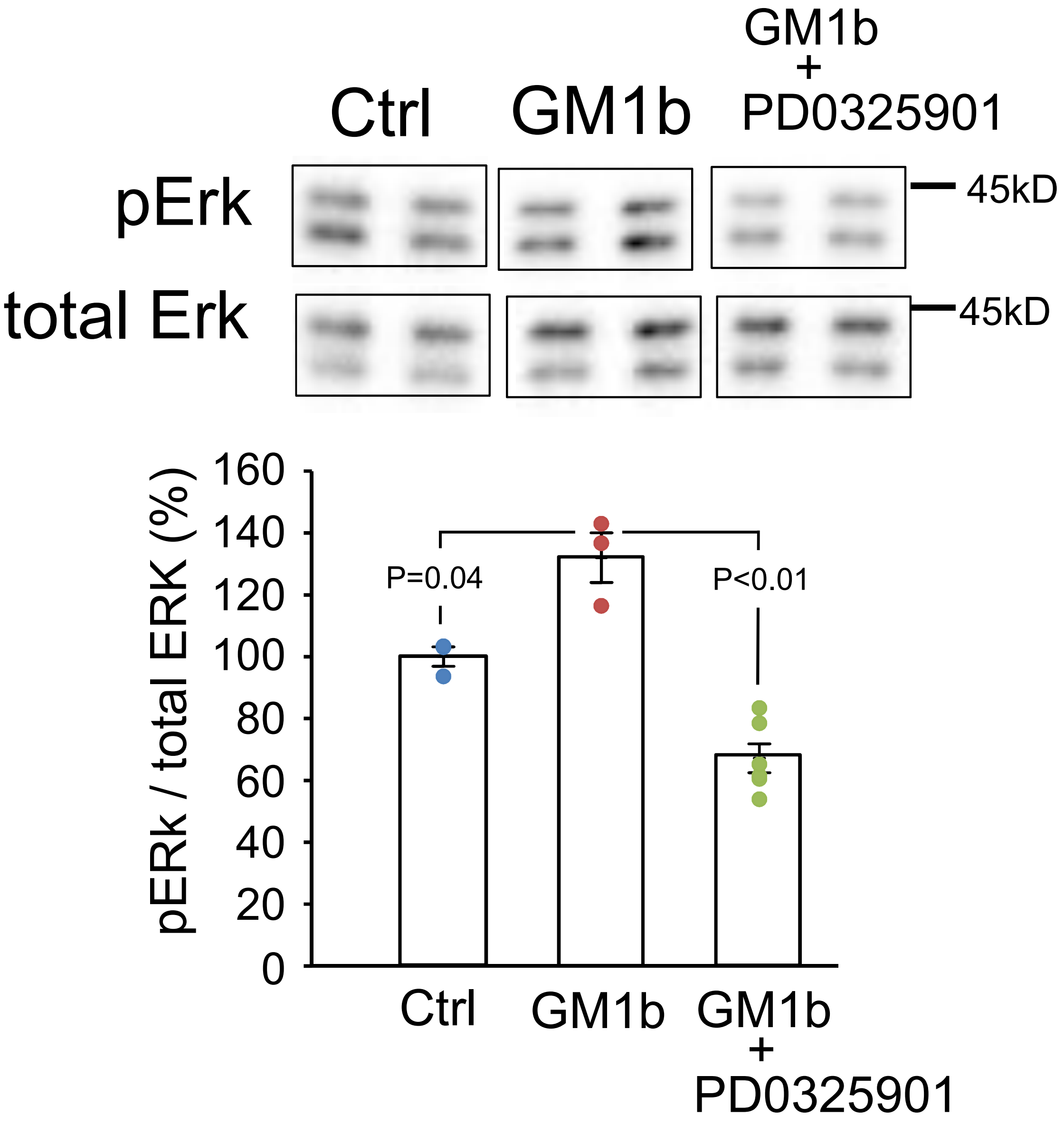

Supplementary Fig. 20

A model of P6-mediated suppression of metastasis

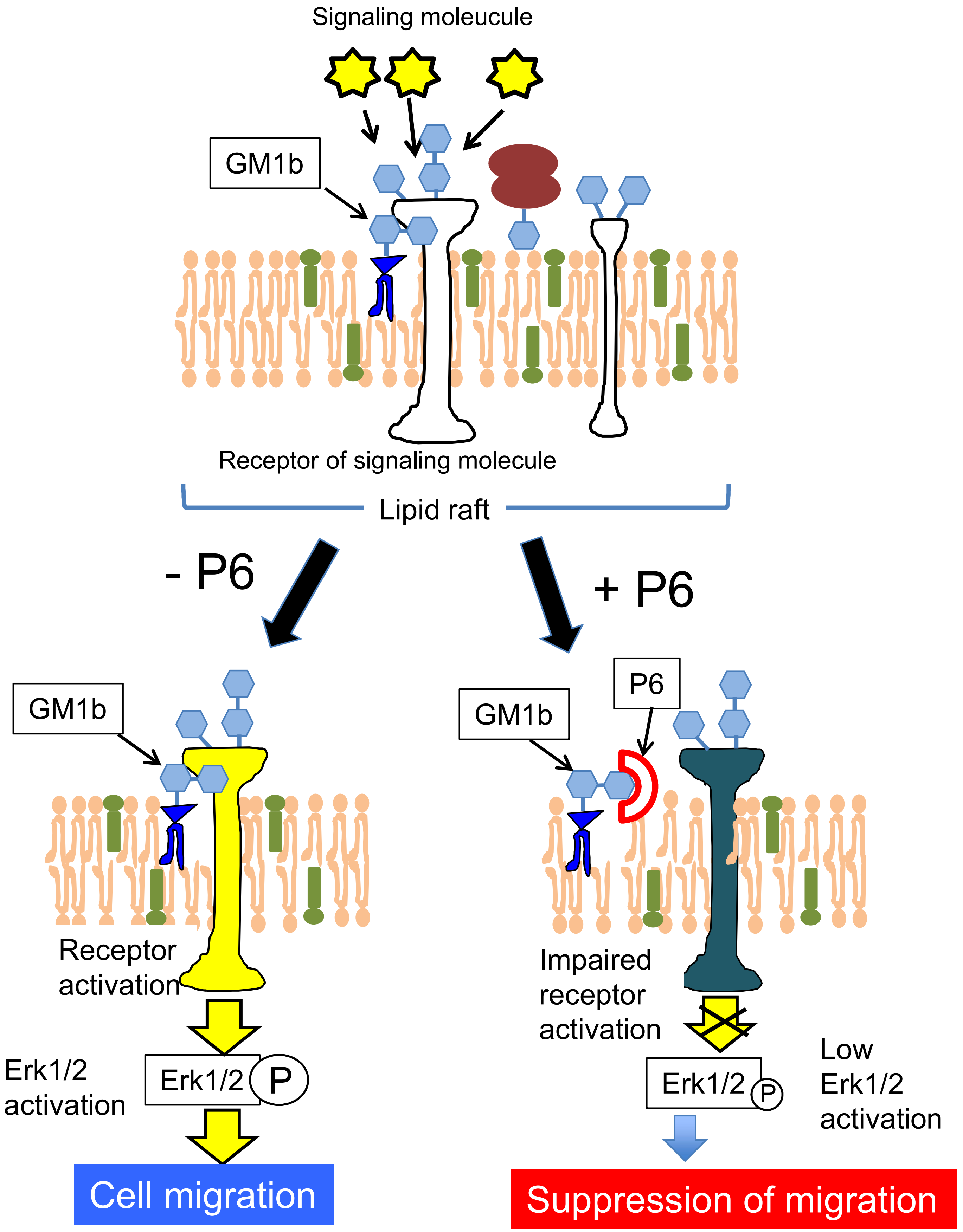

Supplementary Fig.21

Unedited/uncropped western blot gels for Fig.1f

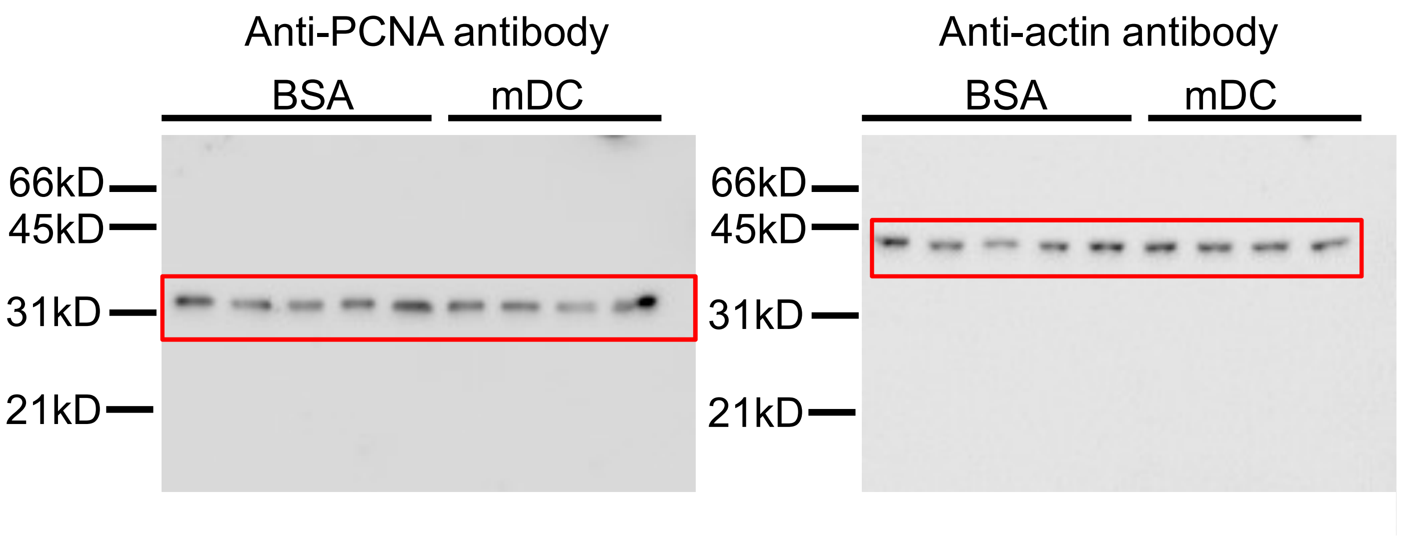

Supplementary Fig. 22

Unedited/uncropped western blot gels for Fig.3b

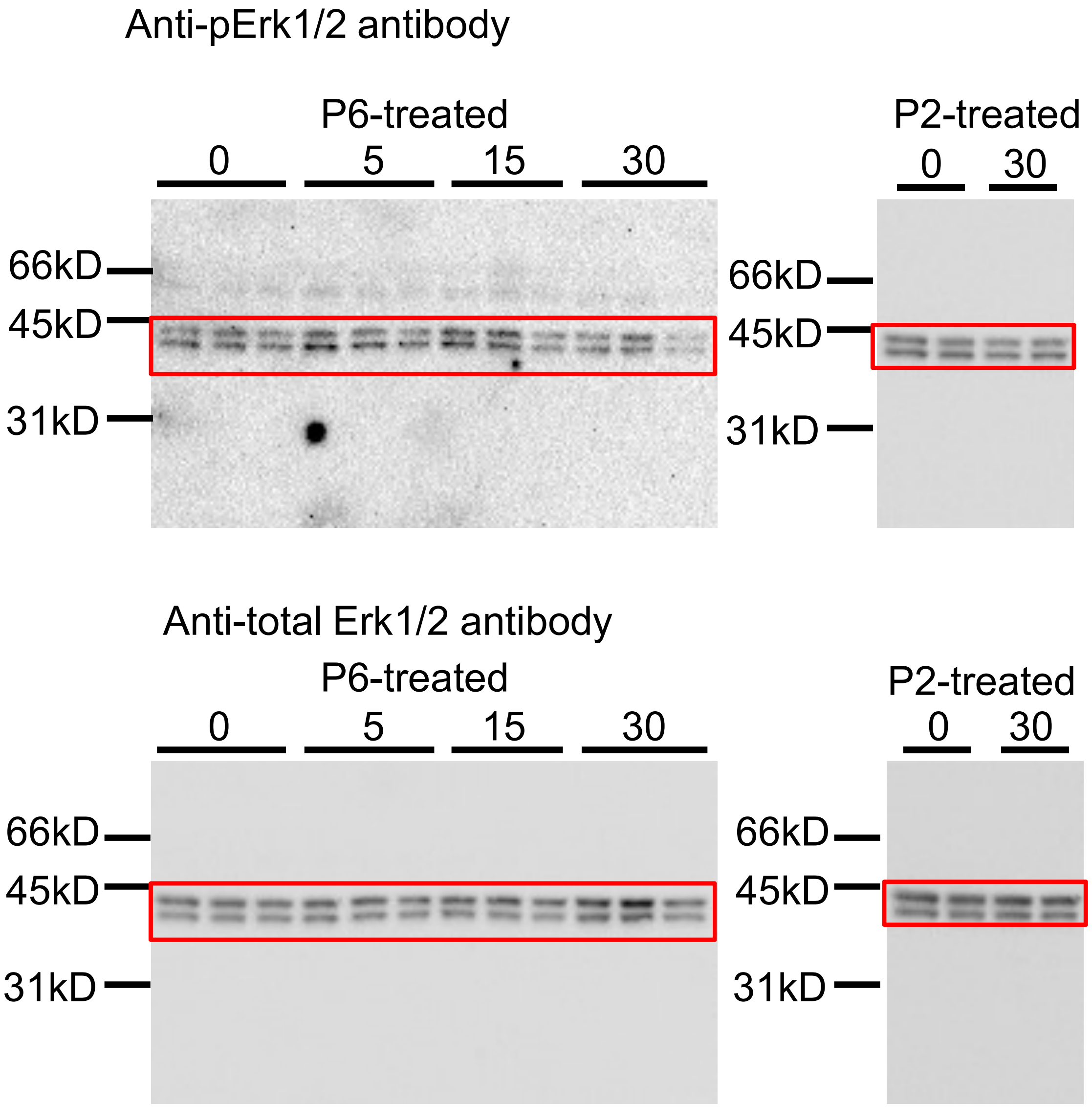

Supplementary Fig. 23

Unedited/uncropped western blot gels for Fig.3d

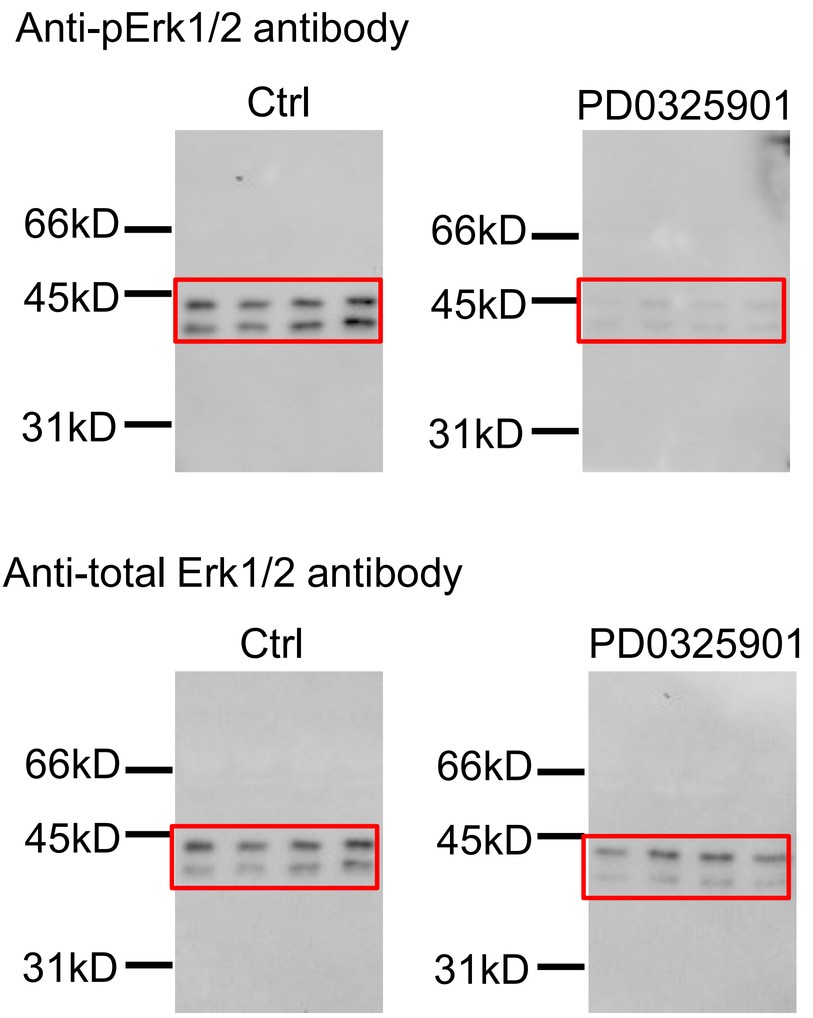

Supplementary Fig. 24

Unedited/uncropped western blot gels for Fig.4f

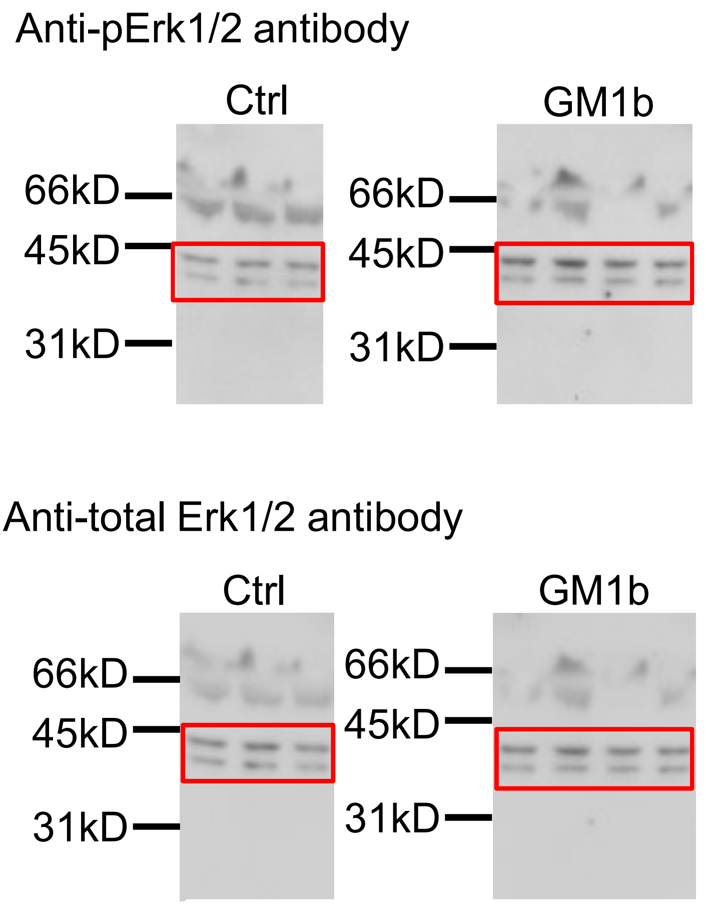

Supplementary Fig. 25

Unedited/uncropped western blot gels for Supplementary Fig.16

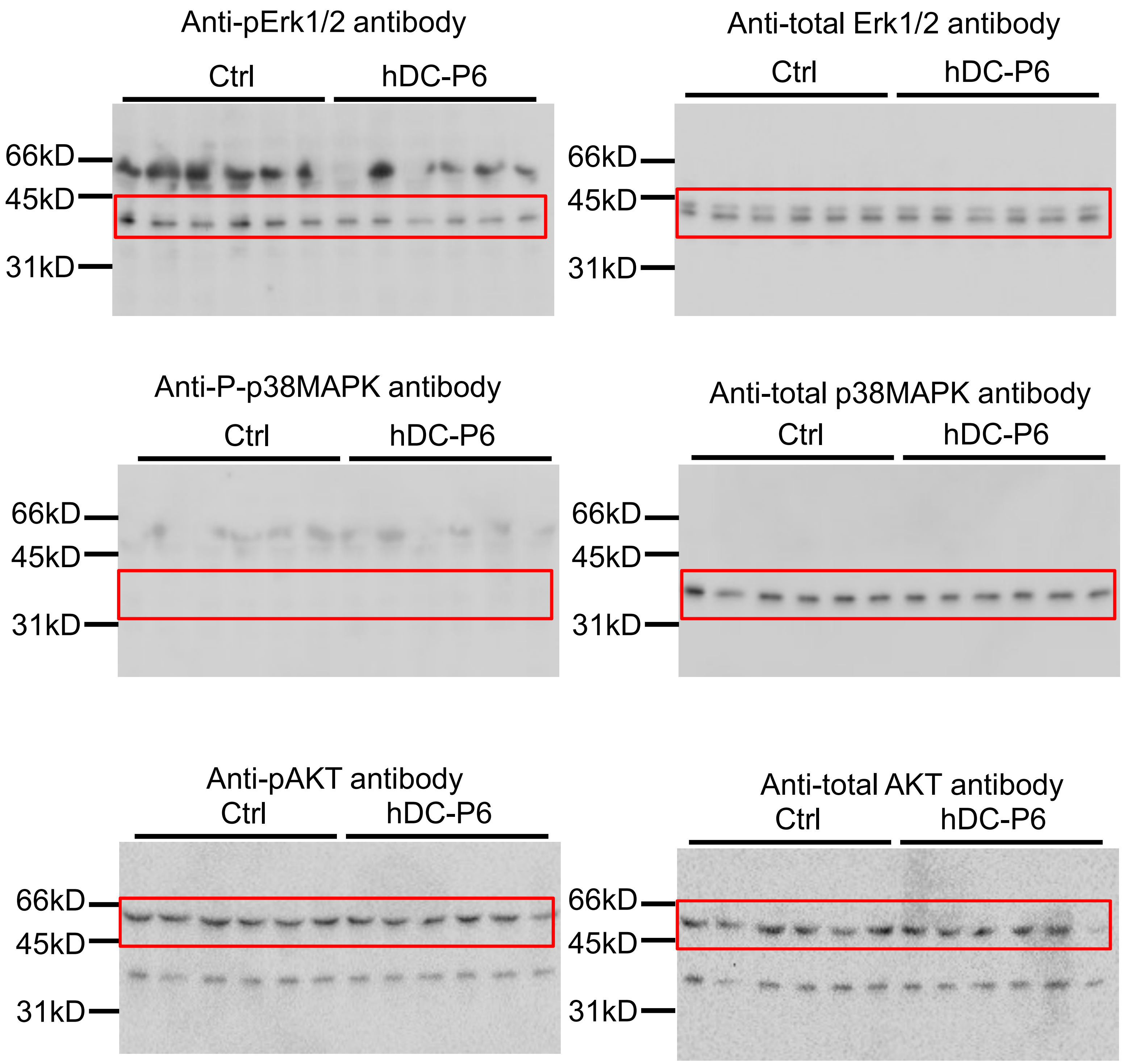

Supplementary Fig. 26

Unedited/uncropped western blot gels for Supplementary Fig.20

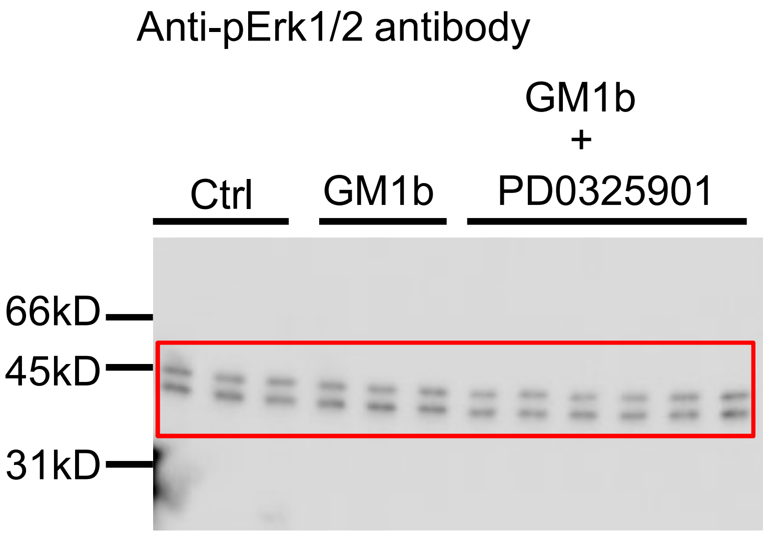

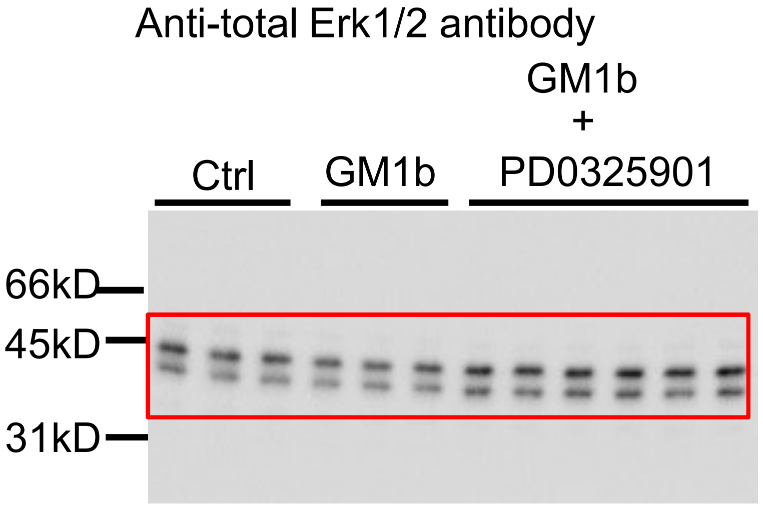

Supplementary Fig. 27
