# Supplementary material for: Dicalcin suppresses invasion and metastasis of mammalian ovarian cancer cells by regulating the ganglioside-Erk1/2 axis
Source: Commun Biol. 2023 Oct 6;6:1015. doi: 10.1038/s42003-023-05324-w (PMC10558574; doi:10.1038/s42003-023-05324-w)
Supplement: Supplementary file 2 — Reporting Summary [file 42003_2023_5324_MOESM2_ESM.pdf]

Corresponding author(s): Naofumi Miwa
Last updated by author(s): Aug 15, 2023

Reporting Summary

Nature Portfolio wishes to improve the reproducibility of the work that we publish. This form provides structure for consistency and transparency in reporting. For further information on Nature Portfolio policies, see our Editorial Policies and the Editorial Policy Checklist.

Statistics

For all statistical analyses, confirm that the following items are present in the figure legend, table legend, main text, or Methods section.

n/a	Confirmed	
☐	☒	The exact sample size (*n*) for each experimental group/condition, given as a discrete number and unit of measurement
☐	☒	A statement on whether measurements were taken from distinct samples or whether the same sample was measured repeatedly
☐	☒	The statistical test(s) used AND whether they are one- or two-sided<br>*Only common tests should be described solely by name; describe more complex techniques in the Methods section.*
☒	☐	A description of all covariates tested
☐	☒	A description of any assumptions or corrections, such as tests of normality and adjustment for multiple comparisons
☐	☒	A full description of the statistical parameters including central tendency (e.g. means) or other basic estimates (e.g. regression coefficient) AND variation (e.g. standard deviation) or associated estimates of uncertainty (e.g. confidence intervals)
☐	☒	For null hypothesis testing, the test statistic (e.g. F, t, r) with confidence intervals, effect sizes, degrees of freedom and P value noted<br>*Give P values as exact values whenever suitable.*
☒	☐	For Bayesian analysis, information on the choice of priors and Markov chain Monte Carlo settings
☒	☐	For hierarchical and complex designs, identification of the appropriate level for tests and full reporting of outcomes
☒	☐	Estimates of effect sizes (e.g. Cohen's *d*, Pearson's *r*), indicating how they were calculated

Our web collection on statistics for biologists contains articles on many of the points above.

Software and code

Policy information about availability of computer code

Data collection	No custom code/software were used in collection of the present data.
Data analysis	Statistical analysis was performed in Excel worksheets.

For manuscripts utilizing custom algorithms or software that are central to the research but not yet described in published literature, software must be made available to editors and reviewers. We strongly encourage code deposition in a community repository (e.g. GitHub). See the Nature Portfolio guidelines for submitting code & software for further information.

Data

Policy information about availability of data

All manuscripts must include a data availability statement. This statement should provide the following information, where applicable:

- Accession codes, unique identifiers, or web links for publicly available datasets
- A description of any restrictions on data availability
- For clinical datasets or third party data, please ensure that the statement adheres to our policy

All data are available from the corresponding author on request.

Research involving human participants, their data, or biological material

Policy information about studies with human participants or human data. See also policy information about sex, gender (identity/presentation), and sexual orientation and race, ethnicity and racism.

Reporting on sex and gender	N/A
Reporting on race, ethnicity, or other socially relevant groupings	N/A
Population characteristics	N/A
Recruitment	N/A
Ethics oversight	N/A

Note that full information on the approval of the study protocol must also be provided in the manuscript.

Field-specific reporting

Please select the one below that is the best fit for your research. If you are not sure, read the appropriate sections before making your selection.

☒ Life sciences ☐ Behavioural & social sciences ☐ Ecological, evolutionary & environmental sciences

For a reference copy of the document with all sections, see nature.com/documents/nr-reporting-summary-flat.pdf

Life sciences study design

All studies must disclose on these points even when the disclosure is negative.

Sample size	For in vivo animal experiment using cancer-bearing mice, we used more than 10 mice per group, and the E value was between 10 and 20 as adequate sample size. For other in vivo experiments, the sample size was based on our previous experiments similar to this in vitro study.
Data exclusions	No data was excluded from analysis.
Replication	Each experiment presented in this study was repeated at least three times.
Randomization	Since randomization is not applicable for the type of our in vitro and in vivo study, data points in the study were not chosen randomly.
Blinding	All quantifications of the raw data were done blind to conditions.

Reporting for specific materials, systems and methods

We require information from authors about some types of materials, experimental systems and methods used in many studies. Here, indicate whether each material, system or method listed is relevant to your study. If you are not sure if a list item applies to your research, read the appropriate section before selecting a response.

Materials & experimental systems

n/a	Involved in the study	
☐	☒	Antibodies
☐	☒	Eukaryotic cell lines
☒	☐	Palaeontology and archaeology
☐	☒	Animals and other organisms
☒	☐	Clinical data
☒	☐	Dual use research of concern
☒	☐	Plants

Methods

n/a	Involved in the study	
☒	☐	ChIP-seq
☐	☒	Flow cytometry
☒	☐	MRI-based neuroimaging

Antibodies

Antibodies used	Anti-phospho-Erk1/2 antibody (Cell signaling, 9101,1:100); anti-Erk1/2 antibody (Santa Cruz, SC-94, 1:5000); anti-phospho Akt (Cell signaling, 4058, 1:100); anti-Akt (Cell signaling, 4691, 1:1000); anti-phospho p38 MAPK (Cell signaling, 9211, 1:100); anti-p38 MAPK (Cell signaling, 9212, 1:1000); anti-actin antibody (Merck, MAB1501, 1:5000); anti-PCNA antibody (Santa Cruz, SC-56, 1:200); anti-

	CD44 antibody (abcam, ab25340, 1:100); Peroxidase-conjugated anti-rabbit IgG (MP BIomedicals, 55691, 1:1000); Peroxidase-conjugated anti-mouse IgG (MP BIomedicals, 55567, 1:1000) Alexa 594-conjugated anti-rabbit IgG (Thermo Fisher Scientific, A11037, 1:1000); Alexa 488-conjugated anti-rabbit IgG (Thermo Fisher Scientific, A11029, 1:1000).
Validation	All antibodies are widely used, well validated commercial products.

Eukaryotic cell lines

Policy information about cell lines and Sex and Gender in Research

Cell line source(s)	Mouse ovarian cancer cell line (OV2944-HM-1, RCB1483, Riken Bioresourse, Tsukuba, Japan); Human ovarian cancer cell line (OVCAR-3, RCB2135, Riken Bioresourse, Tsukuba, Japan), Human prostate cancer cell line (PC-3, RCB2145, Riken Bioresourse, Tsukuba, Japan), SV40-infected immortal mouse normal ovarian epithelial cell line (T-Ag-Mose, JCRB0151, JCRB cell bank, Osaka, Japan)
Authentication	N/A
Mycoplasma contamination	All cell lines were tested negative for microplasma contamination.
Commonly misidentified lines (See ICLAC register)	N/A

Animals and other research organisms

Policy information about studies involving animals; ARRIVE guidelines recommended for reporting animal research, and Sex and Gender in Research

Laboratory animals	Mus musculus, B6C3F1/Crl, Charles river
Wild animals	No wild animals were used in this study.
Reporting on sex	Female mice
Field-collected samples	N/A
Ethics oversight	All animal experiments were approved and in accordance with the animal committee's at Saitama Medical University and Toho University.

Note that full information on the approval of the study protocol must also be provided in the manuscript.

Flow Cytometry

Plots

Confirm that:

☒ The axis labels state the marker and fluorochrome used (e.g. CD4-FITC).

☒ The axis scales are clearly visible. Include numbers along axes only for bottom left plot of group (a 'group' is an analysis of identical markers).

☒ All plots are contour plots with outliers or pseudocolor plots.

☒ A numerical value for number of cells or percentage (with statistics) is provided.

Methodology

Sample preparation	OV2944 cells were transfected with tdTomato (2 mg/100 ml, Invitrogen) using FuGENE HD transfection reagent (Promega). Two days later, cells were dissociated and tdTomato-expressing cells (i.e., fluorescently-labeled cells) were isolated using a FACSAria cell sorter (BD Bioscience) into DMEM with 10%FBS.
Instrument	FACSAria cell sorter (BD Bioscience)
Software	FACSDiva software (BD Bioscience)
Cell population abundance	The population ratio of tdTomato+ OV2944 cells were considerably high.

Gating strategy

The gate was set to to capture the majority of tdTomato+ OV2944 cell. All captured cells were confirmed tdTomato+ cells.

☒ Tick this box to confirm that a figure exemplifying the gating strategy is provided in the Supplementary Information.
